# Clinical outcomes of critical limb ischemia in Buerger disease: A contemporary analysis from an academic referral center in Jordan

**DOI:** 10.1371/journal.pone.0351071

**Published:** 2026-06-10

**Authors:** Qusai Aljarrah, Mohamad Al-Badaineh, Hammam Bani Salman, Nizar R. Alwaqfi, Sohail Bakkar, Mahmoud Megdadi, Khalid A. Kheirallah, Mohammed Z. Allouh

**Affiliations:** 1 Department of General and Vascular Surgery, Faculty of Medicine, Jordan University of Science and Technology, Irbid, Jordan; 2 Department of Surgery, Faculty of Medicine, The Hashemite University, Zarqa, Jordan; 3 Department of Public Health and Community Medicine, Faculty of Medicine, Jordan University of Science and Technology, Irbid, Jordan; 4 Department of Anatomy, College of Medicine and Health Sciences, United Arab Emirates University, Al Ain, United Arab Emirates; Hiroshima University: Hiroshima Daigaku, JAPAN

## Abstract

**Objective:**

Buerger disease (BD), *thromboangiitis obliterans*, is a rare occlusive peripheral arterial disease affecting young smokers. To date, no study has investigated the burden of this disease in Jordan. This study aimed to assess the clinical course and factors associated with adverse outcomes of BD patients with critical limb ischemia (CLI) in Jordan.

**Methods:**

The medical records of all patients with BD who presented with CLI at King Abdullah University Hospital in Jordan between January 2015 and January 2024 were retrospectively retrieved. The epidemiological profile of retrieved cases was collected along with the course of treatment, adverse events, and outcome. Statistical analyses were performed to assess the relationships between patient presentation, treatment course, and outcome.

**Results:**

The study comprised 41 BD patients with Rutherford categories (RC) (IV-VI). The majority were males (95.1%) in their third decade (51.2%). Most patients (92.7%) presented with ischemic ulcerations (RC V, VI). A multivariable regression model revealed that increased C-reactive protein levels (OR = 1.044, 95% CI [1.003, 1.087], p = 0.035), gangrene at presentation (RC VI) (OR = 11.545, 95% CI [1.444, 92.332], p = 0.021), and limb infections at admission (OR = 7.745, 95% CI [1.182, 50.755], p = 0.033) were practical predictors of ending up with major limb amputation.

**Conclusion:**

In Jordan, BD is associated with a high rate of amputation, which imposes considerable financial and social burdens. Prompt recognition, early management, and stringent smoking cessation measures are imperative to slowing disease progression.

## Introduction

Thromboangiitis obliterans, also referred to as “Buerger disease” (BD), is a rare non-atherosclerotic pan-arteritis characterized by segmental thrombotic occlusions of small- and medium-sized arteries and veins of the distal extremities [1–4]. This condition presents a diagnostic challenge when it predominantly affects young adults and is frequently misdiagnosed as musculoskeletal disorders [2,5]. It was initially documented by Felix von Winiwarter in the 19th century and subsequently described by Buerger in 1908 [6].

The prevalence of BD exhibits geographical variation across continents [7,8]. It demonstrates a higher prevalence in regions of Asia and the Middle East and North Africa (MENA) region, where it can account for a substantial proportion of peripheral arterial disease (PAD) in these areas, whereas it shows a lower prevalence in Western countries [6,7,9,10]. Still, the pathophysiology of BD remains obscure and multifactorial, encompassing genetic predisposition, immunopathogenesis, environmental factors, and endothelial dysfunction associated with nicotine exposure [3,6,8,9]. Although smoking is not only significant for disease initiation but is also associated with disease progression, the nature of this relationship remains incompletely elucidated [1,2,8,11].

The manifestations of BD are highly diverse, including joint-related issues occurring before the pre-occlusive phase, superficial venous thrombophlebitis occurring prior to ischemic symptoms, and intermittent claudication in the feet [12,13]. Patients, moreover, may experience critical limb ischemia (CLI), which could lead to a higher probability of amputation compared to those with atherosclerosis [14]. Another characteristic of BD is its unpredictable nature, with symptoms that periodically intensify and subside [15]. Numerous studies have concluded that CLI is considered the most prevalent presentation in BD; more than half of the patients presented with ischemic ulcerations at the time of initial assessment [2,4,9,12,16]. These ulcerations were predominantly observed in the lower extremities [12,13].

Establishing a diagnosis for this disease presents the primary challenge for physicians due to the absence of specific biomarkers or well-identified etiological factors [6,17,18]. Nevertheless, the Shionoya criteria remain the most widely utilized in medical practice to establish the diagnosis of BD [19]. This is attributed to their cost-effectiveness and adaptability to regional healthcare infrastructure, facilitating accessibility in resource-limited settings such as Jordan and enabling seamless integration into clinical practice without necessitating expensive equipment or significant modifications.

Despite its relatively high prevalence in the MENA region, there is a scarcity of published data from this area [20,21]. To date, no study has investigated the burden of this disease in Jordan. This study aimed to highlight the epidemiological and clinical characteristics of BD in Jordan. More importantly, it investigated the main risk factors associated with disease progression and outcomes in the region.

## Methods

### Ethical consideration

This single-center, retrospective cohort study was conducted with approval from the Institutional Review Board at Jordan University of Science and Technology (JUST, approval ref.: Feb2025/179–27). The study protocol adheres to the ethical guidelines of the current version of the Declaration of Helsinki (2024). The requirement for informed consent was waived owing to the retrospective nature of the study.

### Study protocol

Cases were identified retrospectively from the electronic medical records of King Abdullah University Hospital affiliated with JUST between January 2015 and January 2024 by searching the keyword “critical limb ischemia” and then identifying patients with BD using the keyword “Buerger disease.” The diagnosis of BD was based on the Shionoya criteria and characteristic angiographic findings [19,22]. A clinical diagnosis of BD was established only if the patient fulfilled at least four criteria of Shionoya’s recommendations, exhibited typical angiographic findings, and demonstrated radiological absence of atherosclerotic plaques. Limb involvement was determined on the clinical basis of ischemic rest pain or tissue loss, Rutherford categories (RC) (IV-VI) exclusively [23]. Data were extracted between 12 March 2025 and 1 April 2025 from electronic medical records at King Abdullah University Hospital, and comprised demographics, associated comorbidities (diabetes, hypertension, dyslipidemia), RC at diagnosis, baseline laboratory results, clinical and anatomical features, and treatment provided.

**Fig 1:**
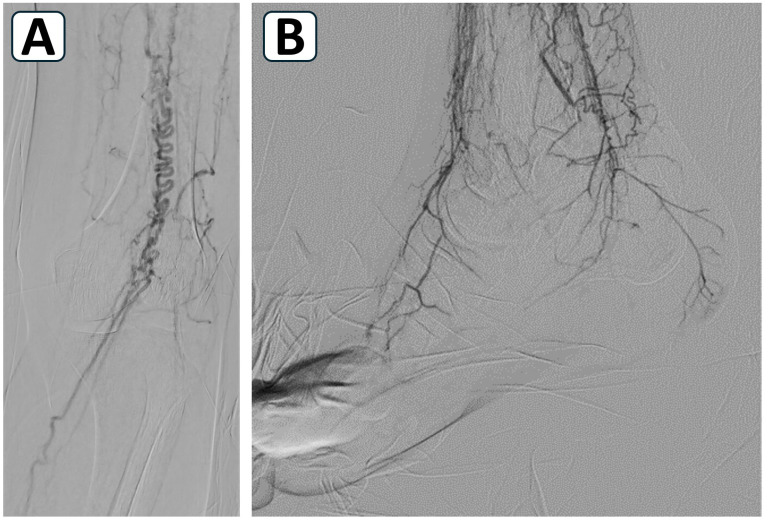
Digital Subtraction Angiographic pattern of Buerger disease in a young male patient. **(A)** Occluded distal femoropopliteal segment with typical corkscrew collaterals and cutoff occlusions. **(B)** The distal tibial vessels of the same patient, with extensive attenuation of the plantar and digital arteries, with compensatory collateral circulation.

Computed tomographic angiography (CTA) was performed in most patients as the initial radiologic examination to corroborate clinical data and delineate the patterns of arterial lesions consistent with BD. Additionally, digital subtraction angiography (DSA) was further employed in 28 patients who underwent an endovascular procedure, since it provides a crucial guide for directing endovascular therapy (**Fig 1**). Multilevel arterial involvement was defined as patients presenting with proximal arterial disease (above knee popliteal (P1 + P2) segment), femoropopliteal lesion or aortoiliac disease, and infrapopliteal (IP) disease. Infrapopliteal involvement encompassed the below-knee popliteal artery (P3 segment) and tibial vessel disease, as determined by angiographic imaging.

Treatment strategies were categorized as either conservative pharmacotherapy or revascularization procedures. The former encompassed antiplatelets, anticoagulants, vasoactive medications, calcium channel blockers, analgesics, and smoking cessation interventions, combined with local wound care. The latter comprised endovascular, surgical, or combined approaches. Additionally, chemical lumbar sympathectomy was performed as an adjunctive treatment in a subset of patients. All patients were consistently advised to avoid exposure to all forms of tobacco. Tissue infection at diagnosis was documented by a positive tissue culture and associated local signs of infection.

The study outcomes encompassed both minor and major amputations. Amputations were categorized as either major limb amputation (MLA), defined as amputation of the lower limb (above the ankle) or upper limb (above the wrist), or minor amputation, defined as amputation of a hand, foot, or any part thereof. Limb salvage was defined as the absence of above-ankle amputation.

Employment status subsequent to disease onset was also investigated. Patients were monitored in the outpatient clinic in accordance with each patient’s clinical status. The most recent follow-up data, including all treatments administered for BD, the presence or absence of limb amputation, and smoking cessation, were recorded and analyzed.

### Statistical analyses

The frequency distribution and percentages were employed to describe the categorical variables examined in this study, whilst mean ± standard deviation was utilized for continuous variables. A chi-squared goodness-of-fit test was conducted to assess whether the observed frequencies differed significantly from the expected distribution. The associations between categorical variables were analyzed using Pearson’s chi-square (χ2) test, while the one-way ANOVA test was performed to determine the significant differences between the continuous variables. Furthermore, to identify the main predictors of MLA in BD patients within the study model, a multivariable ordinal logistic regression analysis was conducted. A P-value of <0.05 was considered statistically significant. Additionally, to ascertain the precise significance in the contingency tables, a post-hoc residual analysis was performed. Finally, Fisher–Freeman–Halton Exact Test was employed to evaluate the association between treatment modality (conservative, open surgical, endovascular, and combined revascularization approaches) and clinical outcome (no amputation, minor amputation, major amputation), given the small sample size and the presence of sparse data across some categories. All statistical analyses were performed using IBM SPSS Statistics software (standard version 29.0.0.0, IBM, Armonk, NY, USA).

## Results

### General and clinical characteristics of the patients

A total of 41 eligible patients were retrospectively identified and included. Missing baseline data were limited to C-reactive protein (CRP) values in 9 patients; all other baseline variables were complete. The majority of patients were males (95.1%). The mean age of all patients was approximately 38.5 years, and more than half (51.2%, p < 0.01) were in their third decade (**Table 1**). The mean duration of the disease from diagnosis was approximately 8 years. About one-third of the patients (29.3%) had associated vascular comorbidities. All patients were ever smokers (current or former smokers), with the majority (90.2%) being heavy smokers (≥ 20 packs/y). The mean duration of smoking was 24.4 ± 10.5 years, with most patients (23/41, 56.1%) having a smoking duration exceeding 20 years. Furthermore, the majority of patients (73.2%) lacked post-secondary education. More than two-third of cases (68.3%) became unemployed subsequent to disease onset (**Table 1**). These findings indicate that the general characteristics of our patient cohort closely align with the typical profile of BD patients, namely heavy-smoking young adult males of low socioeconomic status.

**Table 1:** General characteristics of patients with Buerger disease (thromboangiitis obliterans) at a referral center in Jordan.

Characteristic	Number	Percent (%)
**Mean ± SD**	
**Sex**		
Male	39^↑↑^	95.1
Female	2	4.9
**Mean Age at Diagnosis (y)**	38.4 ± 7.0	
**Age at Diagnosis (Category)**		
20–29 y	3	7.3
30–39 y	21^↑↑^	51.2
40–49 y	16	39.0
50–59 y	1	2.4
**Mean Duration from Diagnosis (y)**	7.9 ± 6.8	
**Duration from Diagnosis**		
≤ 5 y	21	51.2
> 5 y	20	48.8
**Smoking Status**		
Current	33^↑↑^	80.5
Former	8	19.5
Never	0	0
**Mean Smoking Duration (y)**	24.4 ± 10.5	
**Smoking Amount (pack/y)**		
< 20	4	9.8
≥ 20	37^↑↑^	90.2
**Presence of Comorbidities (***diabetes, hypertension, and/or hyperlipidemia*)		
Yes	12	29.3
No	29	70.7
**Laboratory Examination**		
High CRP (>10 mg/L)	24	75.0*
**Education**		
Primary school	9	22.0
Secondary school	21	51.2
Undergraduate degree	9	22.0
Postgraduate degree	2	4.9
**Job Loss**		
Yes	28^↑^	68.3
No	13	31.7
**Mean Hospital Readmission/y**	5.4 ± 5.8	
**Hospital Readmission/y**		
0–5	30^↑↑^	73.2
6–10	5	12.2
11–15	3	7.3
> 15	3	7.3

Abbreviations: CRP, C-reactive protein; SD, standard deviation; y, year.

^↑^ (p < 0.05), ^↑↑^(p < 0.01): significantly higher than expected frequency (χ^2^ goodness of fit test). *Out of 32 patients since there were nine patients without CRP values in their medical records.

The clinical characteristics of BD patients are summarized in **Table 2**. Twenty patients (48.8%) presented with a single limb affected, an additional twenty (48.8%) patients exhibited involvement of two limbs, and only one (2.4%) patient demonstrated the involvement of three limbs. The study encompassed patients with CLI (RC IV-VI). Only three cases (7.3%) suffered from ischemic rest pain alone (RC IV), whilst the majority presented with either non-healing ulcers (25/41, 61.0%, RC V) or gangrenous necrosis (13/41, 31.7%, RC VI). In most cases (32/41, 78.0%), both anterior and posterior tibial arteries were affected. Nearly half of the patients (19/41, 46.3%) exhibited multilevel disease, involving both infrapopliteal and suprapopliteal arteries (**Table 2**).

**Table 2:** Clinical presentations of patients with Buerger disease (thromboangiitis obliterans) at a referral center in Jordan.

	Number	Percent (%)
**Mean ± SD**	
**Number of Limbs Affected**		
1	20	48.8
2	20	48.8
3	1	2.4
**Rutherford Classification**		
Category 4 (rest pain)	3^↓↓^	7.3
Category 5 (ulcer)	25^↑↑^	61.0
Category 6 (gangrene)	13	31.7
**Presence of Ischemic Ulcer**		
Unilateral lower limb	18^↑↑^	43.9
Bilateral lower limb	14	34.1
Upper and unilateral lower limbs	4	9.8
Upper and Bilateral lower limbs	1	2.4
Upper limb alone	1	2.4
None	3	7.3
**Involved Artery**		
Anterior tibial artery	5	12.2
Posterior tibial artery	4	9.8
Both	32^↑↑^	78.0
**Multilevel Disease**		
Yes	19	46.3
No	22	53.7
**Thrombophlebitis**		
Yes	14	34.1
No	27	65.9
**Infection at Admission**		
Yes	13	31.7
No	28	68.3

Abbreviations: SD, standard deviation.

↑↑ (p < 0.01): significantly higher than expected frequency (χ^2^ goodness of fit test).

↓↓ (p < 0.01): significantly lower than expected frequency (χ^2^ goodness of fit test).

### Treatment methods and outcomes

The various treatment modalities and outcomes of patients with BD are summarized in **Table 3**. A total of 8 patients (19.5%) received medical treatment only, while 33 patients (80.5%) underwent interventional revascularization procedures. Only 8 patients (19.5%) were admitted to smoking cessation. Some patients received adjunctive treatments, including intravenous prostaglandin infusion (7.3%) and chemical lumbar sympathectomy (12.2%). Regarding revascularization, there was a significantly higher-than-expected frequency of patients who underwent endovascular revascularization exclusively (19/33, p < 0.05) compared to those who underwent open surgical procedures (5/33) or a combination of endovascular and open revascularization procedures (9/33).

**Table 3:** Treatment methods and outcomes of patients with Buerger disease (thromboangiitis obliterans) at a referral center in Jordan.

	Number	Percent (%)
**Mean ± SD**	
**Treatment Method**		
Medical therapy alone	8	19.5
Revascularization	33	80.5
**Revascularization Procedure**		
Endovascular procedure	19^↑^	46.3
Surgical procedure	5	12.2
Both	9	22.0
**Patient Outcome**		
No amputation	15	36.6
Minor amputation	15	36.6
Major amputation	11	26.8
**Amputation Location**		
Lower limb	24^↑↑^	58.5
Upper limb	1	2.4
Both	1	2.4
No amputations	15	36.6

^↑^(p < 0.05), ^↑↑^(p < 0.01): significantly higher than expected frequency (χ^2^ goodness of fit test).

**Fig 2:**
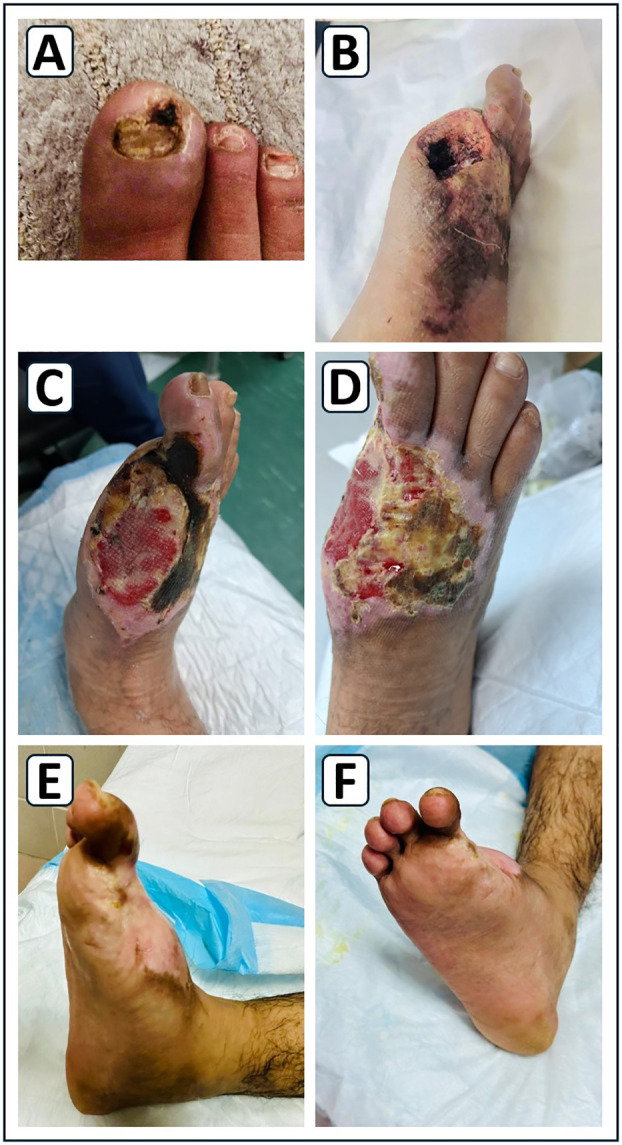
Sequential wound healing progression following amputation of the right great toe in a 27-year-old male diagnosed with Buerger disease. The patient initially presented with an RC V infected ischemic ulcer **(A)**.

In the study population, 15 individuals (36.6%) avoided amputation, while an identical proportion underwent minor limb amputation procedures. A representative example of minor amputation with sequential wound healing in BD is shown in **Fig 2**. The remaining 11 patients (26.8%) underwent major limb amputation (MLA) procedures. Notably, 72.2% of those who underwent MLA (8/11) had previously experienced minor amputation before progressing to MLA.

### Factors associated with patient outcomes

The general factors potentially associated with the outcome of BD patients are summarized in **Table 4**. No statistically significant differences were observed in terms of age and sex on patient outcomes. The duration of the disease was significantly (p < 0.05) associated with the patient’s outcome. Patients who underwent MLA had a significantly (p = 0.048) longer mean duration of the disease compared to patients with minor or no amputations. Moreover, there was a significantly (p < 0.05) higher-than-expected frequency of MLA in patients with disease duration exceeding 5 years (Table 4). Loss of employment (p < 0.01) and frequency of hospital readmission (p < 0.05) were also significantly associated with the outcome of the patients. Patients who underwent minor or MLA were more likely to become unemployed. Furthermore, patients with a readmission rate exceeding three times per year exhibited a significantly (p = 0.017) higher-than-expected frequency of MLA.

**Table 4:** General characteristic factors associated with the outcomes of patients with Buerger disease (thromboangiitis obliterans) at a referral center in Jordan.

Characteristic	No amputation	Minor amputation	Major amputation	p-value*
**Sex**				0.680
Male	14 (35.9)	14 (35.9)	11 (28.2)	
Female	1 (50.0)	1 (50.0)	0 (0.0)	
**Mean Age at Diagnosis (y)**	39.7 ± 7.4	37.2 ± 6.1	38.4 ± 7.9	0.637
**Categorical Age at Diagnosis**				0.879
20–29 y	1 (33.3)	1 (33.3)	1 (33.3)	
30–39 y	7 (33.3)	9 (42.9)	5 (23.8)	
40–49 y	6 (37.5)	5 (31.2)	5 (31.2)	
50–59 y	1 (100.0)	0 (0.0)	0 (0.0)	
**Mean Duration from diagnosis (y)**	5.9 ± 5.5	6.8 ± 5.9	12.1 ± 8.1^↑^	0.048
**Duration from Diagnosis**				0.012
≤ 5 y	10 (52.6)	8 (42.1)	1 (5.3)^↓^	
> 5 y	5 (22.7)	7 (31.8)	10 (45.5)^↑^	
**Smoking Status**				0.229
Current	13 (39.4)	10 (30.3)	10 (30.3)	
Former	2 (25.0)	5 (62.5)	1 (12.5)	
**Smoking Duration**				0.079
≤ 20 y	5 (27.8)	10 (55.6)	3 (16.7)	
> 20 y	10 (43.5)	5 (21.7)	8 (34.8)	
**Presence of Comorbidities**				0.604
Yes	5 (41.7)	3 (25.0)	4 (33.3)	
No	10 (34.5)	12 (41.4)	7 (24.1)	
**Education**				0.495
Primary school	3 (33.3)	2 (22.2)	4 (44.4)	
Secondary school	8 (38.1)	8 (38.1)	5 (23.8)	
Undergraduate degree	4 (44.4)	3 33.3)	2 (22.2)	
Postgraduate degree	0 (0.0)	2 (100.0)	0 (0.0)	
**Family History** ^#^				0.256
Yes	0 (0.0)	2 (50.0)	2 (50.0)	
No	15 (40.5)	13 (35.1)	9 (24.3)	
**Job Loss**				< 0.001
Yes	2 (7.1)	15 (53.6)^↑↑^	11 (39.3)^↑↑^	
No	13 (100.0)^↑↑^	0 (0.0)	0 (0.0)	
**Hospital Readmission**				0.017
Up to 3 per year	11 (50.0)^↑^	9 (40.9)	2 (9.1)	
More than 3 per year	4 (21.1)	6 (31.6)	9 (47.4)^↑^	
**Diagnosis Delay**				0.731
< 1 y	6 (42.9)	4 (28.6)	4 (28.6)	
≥ 1 y	9 (33.3)	11 (40.7)	7 (25.9)	

Abbreviations: p, probability; y, year. ^#^ Family history refers to first degree relatives. Data are presented as mean ± standard deviation for continuous values and as frequency (%) for categorical values. ^↑^(p < 0.05), ^↑↑^(p < 0.01): significantly higher than other groups. ^↓^(p < 0.05): significantly lower than other groups. *p-value was calculated using Pearson’s chi-squared test for categorical variables and one-way ANOVA for continuous variables.

The clinical factors potentially associated with the outcome of patients with BD are summarized in **Table 5**. The serum level of CRP was significantly (p < 0.05) associated with the patient’s outcome. Patients without amputations exhibited significantly (p = 0.025) lower serum CRP levels compared to patients with either minor or major amputations. Furthermore, patients with normal CRP levels demonstrated a significantly (p = 0.041) higher-than-expected frequency of having no amputations compared to patients with elevated (> 10 mg/L) CRP levels. Another significant factor (p < 0.01) associated with the patient’s outcome is the RC at diagnosis. All patients in RC-IV (rest pain) avoided amputations; however, patients in RC-VI (gangrene) exhibited a significantly (p < 0.001) higher-than-expected frequency of MLA. Moreover, the presence of lower limb infection at admission was significantly (p < 0.01) associated with the patient’s outcome. Patients with lower limb infections at admission demonstrated a significantly (p = 0.001) higher-than-expected frequency of MLA compared to other patients, whilst patients without infections at admission exhibited a higher-than-expected frequency of avoiding amputations. The number of affected limbs, involved arteries, level of the disease, and presence of thrombophlebitis were not significantly associated with the outcome of the patients. Additionally, the treatment method, whether conservative (medical only) therapy or conducting a revascularization procedure, was not significantly (p > 0.05) associated with the patient’s outcome (**Table 5**). To further strengthen this analysis, we performed the Fisher–Freeman–Halton exact test to compare all four treatment strategies (conservative, endovascular, open surgical, and combined revascularization) with the amputation outcome. This test did not reveal a statistically significant association (p = 0.324) between the treatment modality and the patient’s outcome.

**Table 5:** Clinical factors associated with the outcomes of patients with Buerger disease (thromboangiitis obliterans) at a referral center in Jordan.

Characteristic	No amputation	Minor amputation	Major amputation	p-value*
**Mean CRP value (mg/L)**	10.7 ± 8.7^↓^	36.0 ± 24.1	43.6 ± 43.3	0.025
**CRP Value**				0.041
Normal CRP (≤ 10 mg/L)	6 (75.0)^↑^	1 (12.5)	1 (12.5)	
High CRP (> 10 mg/L)	6 (25.0)	9 (37.5)	9 (37.5)	
**Number of Affected Limbs**				0.136
1	7 (35.0)	10 (50.0)	3 (15.0)	
2 or more	8 (38.1)	5 (23.8)	8 (38.1)	
**Rutherford Classification**				< 0.001
Category 4 (rest pain)	3 (100.0)^↑^	0 (0.0)	0 (0.0)	
Category 5 (ulcer)	11 (44.0)	12 (48.0)	2 (8.0)	
Category 6 (gangrene)	1 (7.7)	3 (23.1)	9 (69.2)^↑↑^	
**Presence of Ischemic Ulcer on Lower Limb**				0.420
Unilateral lower limb	7 (31.8)	10 (45.5)	5 (22.7)	
Bilateral lower limb	5 (33.3)	4 (26.7)	6 (40.0)	
**Involved arteries**				0.102
Single (ATA or PTA)	6 (66.7)	2 (22.2)	1 (11.1)	
Both (ATA & PTA)	9 (28.1)	13 (40.6)	10 (31.2)	
**Multilevel Disease**				0.405
Yes	6 (31.6)	9 (47.4)	4 (21.1)	
No	9 (40.9)	6 (27.3)	7 (31.8)	
**Thrombophlebitis**				0.793
Yes	6 (42.9)	5 (35.7)	3 (21.4)	
No	9 (33.3)	10 (37.0)	8 (29.6)	
**Infection at Admission**				0.001
Yes	1 (7.7)	4 (30.8)	8 (61.5)^↑↑^	
No	14 (50.0)^↑^	11 (39.3)	3 (10.7)	
**Treatment Method**				0.674
Medical therapy alone	3 (37.5)	2 (25.0)	3 (37.5)	
Revascularization	12 (36.4)	13 (39.4)	8 (24.2)	

Abbreviations: ATA, anterior tibial artery; PTA, posterior tibial artery; p, probability. Data are presented as frequency (%). ^↑^(p < 0.05), ^↑↑^(p < 0.01): significantly higher than other groups. ^↓^(p < 0.05): significantly lower than other groups. *p-value was calculated using Pearson’s chi-squared test.

Finally, a multivariable ordinal logistic regression model was performed to elucidate the primary predictors of MLA as an outcome (**Table 6**). The model incorporated the following predictors: CRP serum level, duration of the disease, RC at diagnosis, and the presence of infection at admission. The analysis revealed that the CRP level was a significant (p < 0.05) predictor of MLA; with each unit increase in the CRP serum level, there was a 1.044-fold greater risk of a MLA outcome (OR = 1.044, 95% CI [1.003, 1.087], p = 0.035). Patients who presented with gangrene (RC-VI) exhibited a significantly (p < 0.05) 11.545-fold greater risk of MLA compared to patients who presented with ischemic ulcers (RC-V) (OR = 11.545, 95% CI [1.444, 92.332], p = 0.021). Moreover, patients who presented with limb infections at the time of admission demonstrated a significantly (p < 0.05) 7.745-fold greater risk of MLA than patients who presented without infections (OR = 7.745, 95% CI [1.182, 50.755], p = 0.033). Lastly, the duration of the disease was not found to be a predictor of MLA outcome (**Table 6**).

**Table 6:** Multivariable ordinal logistic regression model for the prediction of major amputation in patients with Buerger disease (thromboangiitis obliterans) at a referral center in Jordan.

The Predictors	P-value	Odds Ratio	95% CI	
**Lower**	**Upper**
**CRP level**	0.035	1.044	1.003	1.087
**Duration of the Disease**				
> 5 y	0.140	–	0.631	25.795
≤ 5 y	Ref	Ref	Ref	Ref
Rutherford Class*				
Category 6 (gangrene)	0.021	11.545	1.444	92.332
Category 5 (ulcer)	Ref	Ref	Ref	Ref
**Infection at Admission**				
Yes	0.033	7.745	1.182	50.755
No	Ref	Ref	Ref	Ref

Abbreviations: P, probability; CI, confidence interval. The logistic regression model included the four variables reported in this table. *Rutherford category 4 (rest pain) was excluded from the regression model since it only included 3 patients.

## Discussion

To the best of our knowledge, this is the first study to investigate the clinical characteristics and outcomes of Buerger’s disease in Jordan. We found that the cohort was composed mainly of young male heavy smokers who often presented with advanced critical limb ischemia and substantial risk of limb loss. Many patients arrived with ischemic ulcers or gangrene, and progression from minor to major amputation was common. There were three features that could predict major amputation outcome: elevated CRP, gangrene at presentation, and limb infection at admission. Longer disease duration correlated with worse outcomes in unadjusted analyses, although it did not remain an independent predictor after accounting for clinical severity and infection. The disease carried a significant social burden, resulting in frequent hospitalizations and high unemployment rates.

In comparison to other research, this study demonstrated predominantly similar demographic characteristics, with males constituting the majority [24–27]. Female participants comprised 4.9% of the survey respondents, which deviates from recent Western data indicating that females now represent 25% or more of BD cases [1,28]. However, this figure aligns with findings from Asia and the Middle East [24,29–31]. A history of superficial vein thrombosis was observed in 34% of cases in our study, which is consistent with classical descriptions of BD [6,12,32]. Furthermore, our findings corroborate previous studies in that we observed no correlation between limb salvage in BD and patients’ age, sex, extent of cigarette smoking, associated co-morbidities, and level of education [1,11,30,33].

The data indicate that 93% of BD patients in the current study presented with (RC V, VI), which is significantly higher than the 30% in Europe and 45% in Japan, but is consistent with data from Iran [1,16,34–36]. This discrepancy likely reflects late diagnosis rather than a delay in symptom onset, as the intriguing presentation of this disease affecting young adults, subtle early ischemic symptoms, or other disease manifestations can be easily overlooked by patients or attending physicians [4,6]. Our data indicate that 43% of patients who received an early diagnosis (diagnosis delay <1 year) avoided any amputation, whereas only one-third of patients with a delayed diagnosis avoided any amputation. Furthermore, as the majority are day laborers, they often present only when pain becomes intolerable or when associated ischemic ulceration occurs [7]. Alternatively, it may be related to the rapid progression of the disease and its adverse effects on limb viability and patient survival. Olin J. astutely observed that early recognition of BD and timely treatment remain inadequate despite recent advancements in diagnostic tools and therapeutic interventions, and the diagnosis is typically not established until ischemic ulcers manifest in the toes [37].

With regard to anatomy, multi-limb involvement was frequent, and the lower limbs predominated in our series. Multilevel disease was also common. However, neither the presence of multilevel involvement nor the tibial artery distribution (anterior, posterior, or both) showed an association with limb loss. Additionally, we found no difference in the outcomes between patients with single runoff involvement and those with two runoff vessels affected. These findings are consistent with the fluctuating and segmental nature of Buerger’s vasculopathy and suggest that anatomic extent alone is a poor discriminator of prognosis [7,8,32,38].

The prevalence of MLA in BD patients exhibits considerable variation, ranging from 4.3% to 31%, according to studies encompassing individuals with both claudication and CLI and heterogeneous follow-up duration [2,6,12,31,39,40]. Our study found a 27% MLA rate, consistent with Börner and Heidrich, Salimi et al., and Fazeli et al [30,41,42]. However, this rate is notably higher than the 13% reported by Kim et al [29]. This variation may stem from different confounding factors. First, our study’s higher amputation rate may be linked to the severity of disease at presentation; 93% of our cohort had advanced CLI (RC V, VI), aligning with data from developing nations like Iran, in contrast to the 43% with CLI in Kim et al.’s Korean study [27,29,36,42].

Notably, RC at diagnosis was found to be a significant predictor of MLA in our analysis (p < 0.001), which aligns with the published literature, which shows a correlation between RC and disease outcome [1,14,29,37,43,44]. MLA is sometimes offered early to patients with extensive tissue loss and unfeasible revascularization options to decrease the suffering and improve the quality of life [6]. Moreover, BD patients with ischemic ulcers demonstrate a higher propensity for tissue infection [37]. For example, over one-third of our patient cohort presented with limb infections, substantially higher than the 8.5% reported in a previous European study, which also identified infection at presentation as an independent factor for amputation [1]. Additionally, several investigators have demonstrated elevated CRP levels in BD [3,6,45,46]. A recent review identified high CRP levels as a predictor of adverse limb events in PAD patients [47]. This is comparable to our findings, indicating that elevated CRP levels were a positive predictor of amputations in BD patients. We suggest that CRP levels are crucial in assessing amputation risk in BD

Our findings indicate that smoking status and duration did not influence limb salvage rates among the studied cohort. Former smokers were less likely to undergo MLA (12.5%) than current smokers (30.3%), although this difference was not statistically significant (p = 0.229). A similar trend was observed in relation to smoking duration; patients who smoked > 20 years had a 34.8% incidence of MLA, while only 16.7% underwent MLA in patients who smoked ≤20 years (p = 0.079). There are several explanations for this. First, smoking status was self-reported without urine cotinine verification, potentially leading to false claims of cessation. Second, our patients often ceased smoking only when confronted with severe disease progression. Third, some individuals consider non-cigarette tobacco use as cessation, and Jordan lacks smoking cessation education and resources. Fourth, the studied cohort presented with adverse limb events that possibly rendered smoking cessation insufficient to prevent limb loss. Nevertheless, smoking cessation remains the cornerstone of management in BD and is associated with a lower risk of vascular events and amputation. However, in patients who present with advanced critical limb ischemia and established tissue loss, cessation may not immediately eliminate the risk of limb loss because irreversible vascular and tissue damage may already be present.

It is important to emphasize that simple advice to quit smoking, when not accompanied by structured follow-up, behavioral support, and pharmacological treatment, has only limited effectiveness in the literature [48,49]. This may represent one of the factors contributing to the high amputation rate observed in our cohort. Effective smoking cessation usually requires structured programs that incorporate both pharmacological aids and behavioral interventions targeting the physiological and psychological aspects of nicotine dependence. Unfortunately, the healthcare system in Jordan lacks robust smoking cessation clinics, and smoking cessation medications are not generally covered by insurance, which limits patients’ access to the support and treatment necessary for successful cessation.

Evaluating the efficacy of treatments for BD patients presents challenges in data harmonization and is influenced by multiple confounding variables, rendering the comparison of existing data imprecise [15,39]. Consequently, treatment approaches are predominantly tailored based on available resources. The role of revascularization in patients with BD remains a subject of ongoing debate [4,15,24,50]. Our study did not demonstrate a significant difference in limb salvage rates between the revascularization and the conservative treatment groups (75.2% vs. 62.5%, p = 0.674). This is consistent with the findings of previous studies [11,14]. Additionally, this can be explained by several factors specific to BD vasculopathy in our cohort. First, nearly all our patients presented with advanced CLI, in which timely revascularization was not performed to prevent limb loss. Second, many BD cases are diagnosed only after urgent revascularization is already necessary. Third, severe occlusive runoff disease affecting both the anterior and posterior tibial arteries was present in 78% of our cases, leading to the abandonment of the foot in many patients. In fact, revascularization in BD patients is also technically challenging due to the disease’s spastic and thrombogenic nature, compounded by the absence of a standardized, intensive antithrombotic regimen [24,51].

We acknowledge that this study has the inherent limitations of a typical retrospective analysis, including single-center registry data from a small sample, potentially restricting generalizability. Furthermore, consensus regarding BD medical management is lacking. No diagnostic test was used to confirm the absolute cessation of smoking, which is an important limitation of the current analysis. Additionally, follow-up data were often unavailable due to intermittent consultations, with gaps frequently attributable to patients’ low socioeconomic status and health insurance limitations. Therefore, the exact number of patients lost to follow-up could not be determined reliably from the available records. These incomplete and irregular data precluded the application of time-to-event analyses, such as Kaplan–Meier curves for amputation-free survival. Also, Post-revascularization patency rates and interval changes in ischemic lesions, including ulcer healing and gangrene progression or regression, could not be analyzed reliably because routine surveillance duplex imaging was not performed according to a standardized protocol, and follow-up documentation was incomplete and irregular as explained earlier. Finally, the small sample size within treatment groups, especially in the conservative and open surgery categories, reduced the statistical power to identify potential differences in amputation outcomes across treatment strategies. Consequently, the non-significant association observed should be interpreted with caution, and validation in larger cohorts is necessary to draw more definitive conclusions.

## Conclusions

In Jordan, BD is associated with a high rate of MLA and significantly contributes to the financial burden and social impact of job loss. Given the severe consequences of BD, including limb amputation and employment termination, prompt recognition and early management may help reduce progression to advanced ischemia, superimposed infection, and tissue loss. Three clinical features emerged as practical predictors of major amputation in this cohort: elevated CRP, gangrene at presentation, and limb infection at admission. Stringent measures to implement smoking cessation are imperative to prevent the rapid progression of the disease. It remains unclear whether the Middle Eastern populations experience a more pronounced clinical progression of BD or if the issue lies in inadequate diagnosis and treatment.
