# Supplementary material for: Clinical outcomes of critical limb ischemia in Buerger disease: A contemporary analysis from an academic referral center in Jordan
Source: PLoS One. 2026 Jun 10;21(6):e0351071. doi: 10.1371/journal.pone.0351071 (PMC13252764; doi:10.1371/journal.pone.0351071)

PLOS ONE

Clinical Outcomes of Critical Limb Ischemia in Buerger Disease: A Contemporary Analysis from an Academic Referral Center in Jordan

--Manuscript Draft--

**Manuscript Number:**	PONE-D-25-59692
**Article Type:**	Research Article
**Full Title:**	Clinical Outcomes of Critical Limb Ischemia in Buerger Disease: A Contemporary Analysis from an Academic Referral Center in Jordan
**Short Title:**	Critical Limb Ischemia in Buerger Disease
**Corresponding Author:**	Mohammed Z. Allouh<br>United Arab Emirates University<br>Al Ain, UNITED ARAB EMIRATES
**Keywords:**	Arteritis; peripheral arterial disease; thromboangiitis obliterans; limb ischemia; Jordan
**Abstract:**	Objective: Buerger disease (BD), thromboangiitis obliterans, is a rare occlusive peripheral arterial disease affecting young smokers. To date, no study has investigated the burden of this disease in Jordan. This study aimed to assess the clinical course and factors associated with adverse outcomes of BD patients with critical limb ischemia (CLI) in Jordan.<br>Methods: The medical records of all patients with BD who presented with CLI at King Abdullah University Hospital in Jordan between January 2015 and January 2024 were retrospectively retrieved. The epidemiological profile of retrieved cases was collected along with the course of treatment, adverse events, and outcome. Statistical analyses were performed to assess the relationships between patient presentation, treatment course, and outcome.<br>Results: The study comprised 41 BD patients with Rutherford categories (RC) (IV-VI). The majority were males (95.1%) in their third decade (51.2%). Most patients (92.7%) presented with ischemic ulcerations (RC V, VI). A multivariable regression model revealed that increased C-reactive protein levels (OR = 1.044, 95% CI [1.003, 1.087], p = 0.035), gangrene at presentation (RC VI) (OR = 11.545, 95% CI [1.444, 92.332], p = 0.021), and limb infections at admission (OR = 7.745, 95% CI [1.182, 50.755], p = 0.033) were practical predictors of ending up with major limb amputation.<br>Conclusion: In Jordan, BD is associated with a high rate of amputation, which imposes considerable financial and social burdens. Early diagnosis is essential, and stringent measures to implement smoking cessation are imperative to slowing disease progression.
**Order of Authors:**	Qusai Aljarrah
	Mohamad Al-Badaineh
	Hammam Bani Salman
	Nizar R. Alwaqfi
	Sohail Bakkar
	Mahmoud Megdadi
	Khalid A. Kheirallah
	Mohammed Z. Allouh
**Additional Information:**	
**Question**	**Response**
**Financial Disclosure**<br><br>Enter a financial disclosure statement that describes the sources of funding for the	Yes

Powered by Editorial Manager® and ProduXion Manager® from Aries Systems Corporation

work included in this submission. Review the submission guidelines for detailed requirements. View published research articles from *PLOS ONE* for specific examples.<br><br>This statement is required for submission and **will appear in the published article** if the submission is accepted. Please make sure it is accurate.<br><br>**Funded studies**<br>Enter a statement with the following details:<br>• Initials of the authors who received each award<br>• Grant numbers awarded to each author<br>• The full name of each funder<br>• URL of each funder website<br>• Did the sponsors or funders play any role in the study design, data collection and analysis, decision to publish, or preparation of the manuscript?<br><br>Did you receive funding for this work?	
Please add funding details.<br>as follow-up to "**Financial Disclosure**<br><br>Enter a financial disclosure statement that describes the sources of funding for the work included in this submission. Review the submission guidelines for detailed requirements. View published research articles from *PLOS ONE* for specific examples.<br><br>This statement is required for submission and **will appear in the published article** if the submission is accepted. Please make sure it is accurate.	This study was partially supported by a UPAR grant to M. Z. Allouh from the United Arab Emirates University (Grant code: G00004977, Fund no. 12M219).

**Funded studies**<br>Enter a statement with the following details:<br>• Initials of the authors who received each award<br>• Grant numbers awarded to each author<br>• The full name of each funder<br>• URL of each funder website<br>• Did the sponsors or funders play any role in the study design, data collection and analysis, decision to publish, or preparation of the manuscript?<br><br>Did you receive funding for this work?"	
Please select the country of your main research funder (please select carefully as in some cases this is used in fee calculation).<br>as follow-up to "**Financial Disclosure**<br><br>Enter a financial disclosure statement that describes the sources of funding for the work included in this submission. Review the submission guidelines for detailed requirements. View published research articles from *PLOS ONE* for specific examples.<br><br>This statement is required for submission and **will appear in the published article** if the submission is accepted. Please make sure it is accurate.<br><br>**Funded studies**<br>Enter a statement with the following details:<br>• Initials of the authors who received each award<br>• Grant numbers awarded to each author<br>• The full name of each funder<br>• URL of each funder website<br>• Did the sponsors or funders play any role in the study design, data collection and analysis, decision to publish, or preparation of the manuscript?<br><br>Did you receive funding for this work?"	UNITED ARAB EMIRATES - AE
**Competing Interests**	The authors have declared that no competing interests exist.

Use the instructions below to enter a competing interest statement for this submission. On behalf of all authors, disclose any competing interests that could be perceived to bias this work—acknowledging all financial support and any other relevant financial or non-financial competing interests.<br><br>This statement is required for submission and **will appear in the published article** if the submission is accepted. Please make sure it is accurate and that any funding sources listed in your Funding Information later in the submission form are also declared in your Financial Disclosure statement.<br><br>View published research articles from *PLOS ONE* for specific examples.<br><br>**NO authors have competing interests**<br><br>Enter: *The authors have declared that no competing interests exist.*<br><br>**Authors with competing interests**<br><br>Enter competing interest details beginning with this statement:<br><br>*I have read the journal's policy and the authors of this manuscript have the following competing interests: [insert competing interests here]*<br><br>* typeset	
**Ethics Statement**<br><br>Enter an ethics statement for this submission. This statement is required if the study involved:<br><br>• Human participants<br>• Human specimens or tissue<br>• Vertebrate animals or cephalopods<br>• Vertebrate embryos or tissues	This study was approved by the Institutional Review Board (IRB) committee at Jordan University of Science and Technology (JUST, approval Ref.: Feb2025/179-27), and it adheres to the ethical guidelines of the current version of the Declaration of Helsinki (2024). Due to the retrospective nature of the study, the IRB committee at JUST waived the need for written patients' informed consent.

• Field research<br><br>Write "N/A" if the submission does not require an ethics statement.<br><br>General guidance is provided below. Consult the submission guidelines for detailed instructions. **Make sure that all information entered here is included in the Methods section of the manuscript.**	

Powered by Editorial Manager® and ProduXion Manager® from Aries Systems Corporation

**Format for specific study types**<br><br>**Human Subject Research (involving human participants and/or tissue)**<br>• Give the name of the institutional review board or ethics committee that approved the study<br>• Include the approval number and/or a statement indicating approval of this research<br>• Indicate the form of consent obtained (written/oral) or the reason that consent was not obtained (e.g. the data were analyzed anonymously)<br><br>**Animal Research (involving vertebrate animals, embryos or tissues)**<br>• Provide the name of the Institutional Animal Care and Use Committee (IACUC) or other relevant ethics board that reviewed the study protocol, and indicate whether they approved this research or granted a formal waiver of ethical approval<br>• Include an approval number if one was obtained<br>• If the study involved *non-human primates*, add *additional details* about animal welfare and steps taken to ameliorate suffering<br>• If anesthesia, euthanasia, or any kind of animal sacrifice is part of the study, include briefly which substances and/or methods were applied<br><br>**Field Research**<br><br>Include the following details if this study involves the collection of plant, animal, or other materials from a natural setting:<br>• Field permit number<br>• Name of the institution or relevant body that granted permission	
**Data Availability**<br><br>Authors are required to make all data underlying the findings described fully available, without restriction, and from the time of publication. PLOS allows rare exceptions to address legal and ethical concerns. See the PLOS Data Policy and FAQ for detailed information.	Yes - all data are fully available without restriction

A Data Availability Statement describing where the data can be found is required at submission. Your answers to this question constitute the Data Availability Statement and **will be published in the article**, if accepted.<br><br>**Important:** Stating 'data available on request from the author' is not sufficient. If your data are only available upon request, select 'No' for the first question and explain your exceptional situation in the text box.<br><br>Do the authors confirm that all data underlying the findings described in their manuscript are fully available without restriction?	
**Describe where the data may be found in full sentences. If you are copying our sample text, replace any instances of XXX with the appropriate details.**<br><br>• If the data are **held or will be held in a public repository**, include URLs, accession numbers or DOIs. If this information will only be available after acceptance, indicate this by ticking the box below. For example: *All XXX files are available from the XXX database (accession number(s) XXX, XXX.*).<br>• If the data are all contained **within the manuscript and/or Supporting Information files**, enter the following: *All relevant data are within the manuscript and its Supporting Information files.*<br>• If neither of these applies but you are able to provide **details of access elsewhere**, with or without limitations, please do so. For example:<br><br>*Data cannot be shared publicly because of [XXX]. Data are available from the XXX Institutional Data Access / Ethics Committee (contact via XXX) for researchers who meet the criteria for access to confidential data.*<br><br>*The data underlying the results presented in the study are available from (include the name of the third party*	The dataset generated and analysed during the current study is available in the Figshare repository at: https://doi.org/10.6084/m9.figshare.30195802

and contact information or URL).<br>• This text is appropriate if the data are owned by a third party and authors do not have permission to share the data.<br><br>* typeset	
Additional data availability information:	

Powered by Editorial Manager® and ProduXion Manager® from Aries Systems Corporation

Manuscript

Click here to access/download;Manuscript;Buerger Disease Manuscript.docx

Full Title

Clinical Outcomes of Critical Limb Ischemia in Buerger Disease: A Contemporary Analysis from an Academic Referral Center in Jordan

Short Title: Critical Limb Ischemia in Buerger Disease

Authors

Qusai Aljarrah[1]*, Mohamad Al-Badaineh[1], Hammam Bani Salman[1], Nizar R. Alwaqfi[1], Sohail Bakkar[2], Mahmoud Megdadi[3], Khalid A. Kheirallah[3], Mohammed Z. Allouh[4]*

Affiliations

[1] Department of General and Vascular Surgery, Faculty of Medicine, Jordan University of Science and Technology, Irbid-22110, Jordan.

[2] Department of Surgery, Faculty of Medicine, The Hashemite University, Zarqa-13133, Jordan.

[3] Department of Public Health and Community Medicine, Faculty of Medicine, Jordan University of Science and Technology, Irbid- 22110, Jordan.

[4] Department of Anatomy, College of Medicine and Health Sciences, United Arab Emirates University, Al Ain-15551, UAE.

***Corresponding authors**

Qusai Aljarrah

Department of General and Vascular Surgery, Faculty of Medicine, Jordan University of Science and Technology, Irbid, 22110, Jordan.

Tel +962 77 559 3131
Email: qmaljarrah@just.edu.jo
ORCID iD: https://orcid.org/0000-0003-0234-423X

Mohammed Z. Allouh
Department of Anatomy, College of Medicine and Health Sciences, United Arab Emirates University, Al Ain, 15551, UAE.
Tel +971 3 713 7551
Email: m_allouh@uaeu.ac.ae
ORCID iD: https://orcid.org/0000-0003-0105-6260

Abstract

Objective: Buerger disease (BD), *thromboangiitis obliterans*, is a rare occlusive peripheral arterial disease affecting young smokers. To date, no study has investigated the burden of this disease in Jordan. This study aimed to assess the clinical course and factors associated with adverse outcomes of BD patients with critical limb ischemia (CLI) in Jordan.

Methods: The medical records of all patients with BD who presented with CLI at King Abdullah University Hospital in Jordan between January 2015 and January 2024 were retrospectively retrieved. The epidemiological profile of retrieved cases was collected along with the course of treatment, adverse events, and outcome. Statistical analyses were performed to assess the relationships between patient presentation, treatment course, and outcome.

Results: The study comprised 41 BD patients with Rutherford categories (RC) (IV-VI). The majority were males (95.1%) in their third decade (51.2%). Most patients (92.7%) presented with ischemic ulcerations (RC V, VI). A multivariable regression model revealed that increased C-reactive protein levels (OR = 1.044, 95% CI [1.003, 1.087], $p = 0.035$), gangrene at presentation (RC VI) (OR = 11.545, 95% CI [1.444, 92.332], $p = 0.021$), and limb infections at admission (OR = 7.745, 95% CI [1.182, 50.755], $p = 0.033$) were practical predictors of ending up with major limb amputation.

Conclusion: In Jordan, BD is associated with a high rate of amputation, which imposes considerable financial and social burdens. Early diagnosis is essential, and stringent measures to implement smoking cessation are imperative to slowing disease progression.

Keywords

Arteritis, peripheral arterial disease, thromboangiitis obliterans, limb ischemia, Jordan

Introduction

Thromboangiitis obliterans, also referred to as "Buerger disease" (BD), is a rare non-atherosclerotic pan-arteritis characterized by segmental thrombotic occlusions of small- and medium-sized arteries and veins of the distal extremities [1–4]. This condition presents a diagnostic challenge when it predominantly affects young adults and is frequently misdiagnosed as musculoskeletal disorders [2,5]. It was initially documented by Felix von Winiwarter in the 19th century and subsequently described by Buerger in 1908 [6].

The prevalence of BD exhibits geographical variation across continents [7,8]. It demonstrates a higher prevalence in regions of Asia and the Middle East and North Africa (MENA) region, where it can account for a substantial proportion of peripheral arterial disease (PAD) in these areas, whereas it shows a lower prevalence in Western countries [6,7,9,10]. Still, the pathophysiology of BD remains obscure and multifactorial, encompassing genetic predisposition, immunopathogenesis, environmental factors, and endothelial dysfunction associated with nicotine exposure [3,6,8,9]. Although smoking is not only significant for disease initiation but is also associated with disease progression, the nature of this relationship remains incompletely elucidated [1,2,8,11].

The manifestations of BD are highly diverse, including joint-related issues occurring before the pre-occlusive phase, superficial venous thrombophlebitis occurring prior to ischemic symptoms, and intermittent claudication in the feet [12,13]. Patients, moreover, may experience critical limb ischemia (CLI), which could lead to a higher probability of amputation compared to those with atherosclerosis [14]. Another characteristic of BD is its unpredictable nature, with symptoms that

periodically intensify and subside [15]. Numerous studies have concluded that CLI is considered the most prevalent presentation in BD; more than half of the patients presented with ischemic ulcerations at the time of initial assessment [2,4,9,12,16]. These ulcerations were predominantly observed in the lower extremities [12,13].

Establishing a diagnosis for this disease presents the primary challenge for physicians due to the absence of specific biomarkers or well-identified etiological factors [6,17,18]. Nevertheless, the Shionoya criteria remain the most widely utilized in medical practice to establish the diagnosis of BD [19]. This is attributed to their cost-effectiveness and adaptability to regional healthcare infrastructure, facilitating accessibility in resource-limited settings such as Jordan and enabling seamless integration into clinical practice without necessitating expensive equipment or significant modifications.

Despite its relatively high prevalence in the MENA region, there is a scarcity of published data from this area [20,21]. To date, no study has investigated the burden of this disease in Jordan. This study aimed to highlight the epidemiological and clinical characteristics of BD in Jordan. More importantly, it investigated the main risk factors associated with disease progression and outcomes in the region.

Methods

Study protocol

This cross-sectional, single-center, retrospective study was conducted with approval from the Institutional Review Board at Jordan University of Science and Technology (JUST, approval

ref.: Feb2025/179-27). The study protocol adheres to the ethical guidelines of the current version of the Declaration of Helsinki (2024). The requirement for informed consent was waived owing to the retrospective nature of the study. The research encompassed all patients diagnosed with BD between January 2015 and January 2024, based on the Shionoya criteria and characteristic angiographic findings [22]. A clinical diagnosis of BD was established only if the patient fulfilled at least four criteria of Shionoya's recommendations, exhibited typical angiographic findings, and demonstrated an absence of atherosclerotic plaques. Limb involvement was determined on the clinical basis of ischemic rest pain or tissue loss, Rutherford categories (RC) (IV-VI) exclusively [23]. Data were extracted between 12 March 2025 and 1 April 2025 from electronic medical records at King Abdullah University Hospital affiliated with JUST, and comprised demographics, associated comorbidities (diabetes, hypertension, dyslipidemia), RC at diagnosis, baseline laboratory results, clinical and anatomical features, and treatment provided.

Computed tomographic angiography (CTA) was performed in most patients as the initial radiologic examination to corroborate clinical data and delineate the patterns of arterial lesions consistent with BD. Additionally, digital subtraction angiography (DSA) was further employed in 28 patients who underwent an endovascular procedure, since it provides a crucial guide for directing endovascular therapy (**Figure 1**). Multilevel arterial involvement was defined as patients presenting with proximal arterial disease (above knee popliteal (P1+P2) segment), femoropopliteal lesion or aortoiliac disease, and infrapopliteal (IP) disease. Infrapopliteal involvement encompassed the below-knee popliteal artery (P3 segment) and tibial vessel disease, as determined by angiographic imaging.

Treatment strategies were categorized as either conservative pharmacotherapy or revascularization procedures. The former encompassed antiplatelets, anticoagulants, vasoactive medications, calcium channel blockers, analgesics, and smoking cessation interventions, combined with local wound care. The latter comprised endovascular, surgical, or combined approaches. Additionally, chemical lumbar sympathectomy was performed as an adjunctive treatment in a subset of patients. All patients were consistently advised to avoid exposure to all forms of tobacco. Tissue infection at diagnosis was documented by a positive tissue culture and associated local signs of infection.

The study outcomes encompassed both minor and major amputations. Amputations were categorized as either major limb amputation (MLA), defined as amputation of the lower limb (above the ankle) or upper limb (above the wrist), or minor amputation, defined as amputation of a hand, foot, or any part thereof. Limb salvage was defined as the absence of above-ankle amputation.

Employment status subsequent to disease onset was also investigated. Patients were monitored in the outpatient clinic in accordance with each patient's clinical status. The most recent follow-up data, including all treatments administered for BD, the presence or absence of limb amputation, and smoking cessation, were recorded and analyzed.

Statistical analyses

The frequency distribution and percentages were employed to describe the categorical variables examined in this study, whilst mean ± standard deviation was utilized for continuous variables. A

chi-squared goodness-of-fit test was conducted to assess whether the observed frequencies differed significantly from the expected distribution. The associations between categorical variables were analyzed using Pearson's chi-square ($\chi2$) test, while the one-way ANOVA test was performed to determine the significant differences between the continuous variables. Furthermore, to identify the main predictors of MLA in BD patients within the study model, a multivariable ordinal logistic regression analysis was conducted. A P-value of <0.05 was considered statistically significant. Additionally, to ascertain the precise significance in the contingency tables, a post-hoc residual analysis was performed. Finally, Fisher–Freeman–Halton Exact Test was employed to evaluate the association between treatment modality (conservative, open surgical, endovascular, and combined revascularization approaches) and clinical outcome (no amputation, minor amputation, major amputation), given the small sample size and the presence of sparse data across some categories. All statistical analyses were performed using IBM SPSS Statistics software (standard version 29.0.0.0, IBM, Armonk, NY, USA).

Results

General and clinical characteristics of the patients

The study comprised 41 BD patients, the majority of whom were males (95.1%). The mean age of all patients was approximately 38.5 years, and more than half (51.2%, $p < 0.01$) were in their third decade (**Table 1**). The mean duration of the disease from diagnosis was approximately 8 years. About one-third of the patients (29.3%) had associated vascular comorbidities. All patients were ever smokers (current or former smokers), with the majority (90.2%) being heavy smokers (≥ 20 packs/y). The mean duration of smoking was 24.4 ± 10.5 years, with most patients (23/41, 56.1%) having a smoking duration exceeding 20 years. Furthermore, the majority of patients

(73.2%) lacked post-secondary education. More than two-third of cases (68.3%) became unemployed subsequent to disease onset (**Table 1**). These findings indicate that the general characteristics of our patient cohort closely align with the typical profile of BD patients, namely heavy-smoking young adult males of low socioeconomic status.

The clinical characteristics of BD patients are summarized in **Table 2**. Twenty patients (48.8%) presented with a single limb affected, an additional twenty (48.8%) patients exhibited involvement of two limbs, and only one (2.4%) patient demonstrated the involvement of three limbs. The study encompassed patients with CLI (RC IV-VI). Only three cases (7.3%) suffered from ischemic rest pain alone (RC IV), whilst the majority presented with either non-healing ulcers (25/41, 61.0%, RC V) or gangrenous necrosis (13/41, 31.7%, RC VI). In most cases (32/41, 78.0%), both anterior and posterior tibial arteries were affected. Nearly half of the patients (19/41, 46.3%) exhibited multilevel disease, involving both infrapopliteal and suprapopliteal arteries (**Table 2**).

Treatment methods and outcomes

The various treatment modalities and outcomes of patients with BD are summarized in **Table 3**. A total of 8 patients (19.5%) received medical treatment only, while 33 patients (80.5%) underwent interventional revascularization procedures. Only 8 patients (19.5%) were admitted to smoking cessation. Some patients received adjunctive treatments, including intravenous prostaglandin infusion (7.3%) and chemical lumbar sympathectomy (12.2%). Regarding revascularization, there was a significantly higher-than-expected frequency of patients who underwent endovascular revascularization exclusively (19/33, $p < 0.05$) compared to those who

underwent open surgical procedures (5/33) or a combination of endovascular and open revascularization procedures (9/33).

In the study population, 15 individuals (36.6%) avoided amputation, while an identical proportion underwent minor limb amputation procedures. A representative example of minor amputation with sequential wound healing in BD is shown in **Figure 2**. The remaining 11 patients (26.8%) underwent major limb amputation (MLA) procedures. Notably, 72.2% of those who underwent MLA (8/11) had previously experienced minor amputation before progressing to MLA.

Factors associated with patient outcomes

The general factors potentially associated with the outcome of BD patients are summarized in **Table 4**. No statistically significant differences were observed in terms of age and sex on patient outcomes. The duration of the disease was significantly ($p < 0.05$) associated with the patient's outcome. Patients who underwent MLA had a significantly ($p = 0.048$) longer mean duration of the disease compared to patients with minor or no amputations. Moreover, there was a significantly ($p < 0.05$) higher-than-expected frequency of MLA in patients with disease duration exceeding 5 years (Table 4). Loss of employment ($p < 0.01$) and frequency of hospital readmission ($p < 0.05$) were also significantly associated with the outcome of the patients. Patients who underwent minor or MLA were more likely to become unemployed. Furthermore, patients with a readmission rate exceeding three times per year exhibited a significantly ($p = 0.017$) higher-than-expected frequency of MLA.

The clinical factors potentially associated with the outcome of patients with BD are summarized in **Table 5**. The serum level of C-reactive protein (CRP) was significantly ($p < 0.05$) associated with the patient's outcome. Patients without amputations exhibited significantly ($p = 0.025$) lower serum CRP levels compared to patients with either minor or major amputations. Furthermore, patients with normal CRP levels demonstrated a significantly ($p = 0.041$) higher-than-expected frequency of having no amputations compared to patients with elevated (> 10 mg/L) CRP levels. Another significant factor ($p < 0.01$) associated with the patient's outcome is the RC at diagnosis. All patients in RC-IV (rest pain) avoided amputations; however, patients in RC-VI (gangrene) exhibited a significantly ($p < 0.001$) higher-than-expected frequency of MLA. Moreover, the presence of lower limb infection at admission was significantly ($p < 0.01$) associated with the patient's outcome. Patients with lower limb infections at admission demonstrated a significantly ($p = 0.001$) higher-than-expected frequency of MLA compared to other patients, whilst patients without infections at admission exhibited a higher-than-expected frequency of avoiding amputations. The number of affected limbs, involved arteries, level of the disease, and presence of thrombophlebitis were not significantly associated with the outcome of the patients. Additionally, the treatment method, whether conservative (medical only) therapy or conducting a revascularization procedure, was not significantly ($p > 0.05$) associated with the patient's outcome (**Table 5**). To further strengthen this analysis, we performed the Fisher–Freeman–Halton exact test to compare all four treatment strategies (conservative, endovascular, open surgical, and combined revascularization) with the amputation outcome. This test did not reveal a statistically significant association ($p = 0.324$) between the treatment modality and the patient’s outcome.

Finally, a multivariable ordinal logistic regression model was performed to elucidate the primary predictors of MLA as an outcome (**Table 6**). The model incorporated the following predictors: CRP serum level, duration of the disease, RC at diagnosis, and the presence of infection at admission. The analysis revealed that the CRP level was a significant ($p < 0.05$) predictor of MLA; with each unit increase in the CRP serum level, there was a 1.044-fold greater risk of a MLA outcome (OR = 1.044, 95% CI [1.003, 1.087], p = 0.035). Patients who presented with gangrene (RC-VI) exhibited a significantly ($p < 0.05$) 11.545-fold greater risk of MLA compared to patients who presented with ischemic ulcers (RC-V) (OR = 11.545, 95% CI [1.444, 92.332], p = 0.021). Moreover, patients who presented with limb infections at the time of admission demonstrated a significantly ($p < 0.05$) 7.745-fold greater risk of MLA than patients who presented without infections (OR = 7.745, 95% CI [1.182, 50.755], p = 0.033). Lastly, the duration of the disease was not found to be a predictor of MLA outcome (**Table 6**).

Discussion

To the best of our knowledge, this is the first study to investigate the clinical characteristics and outcomes of Buerger's disease in Jordan. We found that the cohort was composed mainly of young male heavy smokers who often presented with advanced critical limb ischemia and substantial risk of limb loss. Many patients arrived with ischemic ulcers or gangrene, and progression from minor to major amputation was common. There were three features that could predict major amputation outcome: elevated CRP, gangrene at presentation, and limb infection at admission. Longer disease duration correlated with worse outcomes in unadjusted analyses, although it did not remain an independent predictor after accounting for clinical severity and infection. The disease carried a significant social burden, resulting in frequent hospitalizations

and high unemployment rates.

In comparison to other research, this study demonstrated predominantly similar demographic characteristics, with males constituting the majority [24–27]. Female participants comprised 4.9% of the survey respondents, which deviates from recent Western data indicating that females now represent 25% or more of BD cases [1,28]. However, this figure aligns with findings from Asia and the Middle East [24,29–31]. A history of superficial vein thrombosis was observed in 34% of cases in our study, which is consistent with classical descriptions of BD [6,12,32]. Furthermore, our findings corroborate previous studies in that we observed no correlation between limb salvage in BD and patients' age, sex, extent of cigarette smoking, associated co-morbidities, and level of education [1,11,30,33].

The data indicate that 93% of BD patients in the current study presented with (RC V, VI), which is significantly higher than the 30% in Europe and 45% in Japan, but is consistent with data from Iran [1,16,34–36]. This discrepancy likely reflects late diagnosis rather than a delay in symptom onset, as the intriguing presentation of this disease affecting young adults, subtle early ischemic symptoms, or other disease manifestations can be easily overlooked by patients or attending physicians [4,6]. Our data indicate that 43% of patients who received an early diagnosis (diagnosis delay <1 year) avoided any amputation, whereas only one-third of patients with a delayed diagnosis avoided any amputation. Furthermore, as the majority are day laborers, they often present only when pain becomes intolerable or when associated ischemic ulceration occurs [7]. Alternatively, it may be related to the rapid progression of the disease and its adverse effects on limb viability and patient survival. Olin J. astutely observed that early recognition of BD and

timely treatment remain inadequate despite recent advancements in diagnostic tools and therapeutic interventions, and the diagnosis is typically not established until ischemic ulcers manifest in the toes [37].

With regard to anatomy, multi-limb involvement was frequent, and the lower limbs predominated in our series. Multilevel disease was also common. However, neither the presence of multilevel involvement nor the tibial artery distribution (anterior, posterior, or both) showed an association with limb loss. Additionally, we found no difference in the outcomes between patients with single runoff involvement and those with two runoff vessels affected. These findings are consistent with the fluctuating and segmental nature of Buerger's vasculopathy and suggest that anatomic extent alone is a poor discriminator of prognosis [7,8,32,38].

The prevalence of MLA in BD patients exhibits considerable variation, ranging from 4.3% to 31%, according to studies encompassing individuals with both claudication and CLI and heterogeneous follow-up duration [2,6,12,31,39,40]. Our study found a 27% MLA rate, consistent with Börner and Heidrich, Salimi et al., and Fazeli et al [30,41,42]. However, this rate is notably higher than the 13% reported by Kim et al [29]. This variation may stem from different confounding factors. First, our study's higher amputation rate may be linked to the severity of disease at presentation; 93% of our cohort had advanced CLI (RC V, VI), aligning with data from developing nations like Iran, in contrast to the 43% with CLI in Kim et al.'s Korean study [27,29,36,42].

Notably, RC at diagnosis was found to be a significant predictor of MLA in our analysis

(p<0.001), which aligns with the published literature, which shows a correlation between RC and disease outcome [1,14,29,37,43,44]. MLA is sometimes offered early to patients with extensive tissue loss and unfeasible revascularization options to decrease the suffering and improve the quality of life [6]. Moreover, BD patients with ischemic ulcers demonstrate a higher propensity for tissue infection [37]. For example, over one-third of our patient cohort presented with limb infections, substantially higher than the 8.5% reported in a previous European study, which also identified infection at presentation as an independent factor for amputation [1]. Additionally, several investigators have demonstrated elevated CRP levels in BD [3,6,45,46]. A recent review identified high CRP levels as a predictor of adverse limb events in PAD patients [47]. This is comparable to our findings, indicating that elevated CRP levels were a positive predictor of amputations in BD patients. We suggest that CRP levels are crucial in assessing amputation risk in BD

Our findings indicate that smoking status and duration did not influence limb salvage rates among the studied cohort. Former smokers were less likely to undergo MLA (12.5%) than current smokers (30.3%), although this difference was not statistically significant (p= 0.229). A similar trend was observed in relation to smoking duration; patients who smoked > 20 years had a 34.8% incidence of MLA, while only 16.7% underwent MLA in patients who smoked ≤20 years (p=0.079). There are several explanations for this. First, smoking status was self-reported without urine cotinine verification, potentially leading to false claims of cessation. Second, our patients often ceased smoking only when confronted with severe disease progression. Third, some individuals consider non-cigarette tobacco use as cessation, and Jordan lacks smoking cessation education and resources. Fourth, the studied cohort presented with adverse limb events

that possibly rendered smoking cessation insufficient to prevent limb loss. Finally, a previous study indicated that the risk of amputation is only eliminated after an individual has ceased smoking for a period of eight years [40].

Evaluating the efficacy of treatments for BD patients presents challenges in data harmonization and is influenced by multiple confounding variables, rendering the comparison of existing data imprecise [15,39]. Consequently, treatment approaches are predominantly tailored based on available resources. The role of revascularization in patients with BD remains a subject of ongoing debate [4,15,24,48]. Our study did not demonstrate a significant difference in limb salvage rates between the revascularization and the conservative treatment groups (75.2% vs. 62.5%, p=0.674). This is consistent with the findings of previous studies [11,14]. Additionally, this can be explained by several factors specific to BD vasculopathy in our cohort. First, nearly all our patients presented with advanced CLI, in which timely revascularization was not performed to prevent limb loss. Second, many BD cases are diagnosed only after urgent revascularization is already necessary. Third, severe occlusive runoff disease affecting both the anterior and posterior tibial arteries was present in 78% of our cases, leading to the abandonment of the foot in many patients. In fact, revascularization in BD patients is also technically challenging due to the disease's spastic and thrombogenic nature, compounded by the absence of a standardized, intensive antithrombotic regimen [24,49].

We acknowledge that this study has the inherent limitations of a typical retrospective analysis, including single-center registry data from a small sample, potentially restricting generalizability. Furthermore, consensus regarding BD medical management is lacking. No diagnostic test was

used to confirm the absolute cessation of smoking, which is an important limitation of the current analysis. Additionally, follow-up data were often unavailable due to intermittent consultations, with gaps frequently attributable to patients' low socioeconomic status and health insurance limitations. These incomplete and irregular data precluded the application of time-to-event analyses, such as Kaplan–Meier curves for amputation-free survival. Finally, the small sample size within treatment groups, especially in the conservative and open surgery categories, reduced the statistical power to identify potential differences in amputation outcomes across treatment strategies. Consequently, the non-significant association observed should be interpreted with caution, and validation in larger cohorts is necessary to draw more definitive conclusions.

Conclusion

In Jordan, BD is associated with a high rate of MLA and significantly contributes to the financial burden and social impact of job loss. Given the severe consequences of BD, including limb amputation and employment termination, early diagnosis is essential to avert superimposed infection of trophic ulcers. Three clinical features emerged as practical predictors of major amputation in this cohort: elevated CRP, gangrene at presentation, and limb infection at admission. Stringent measures to implement smoking cessation are imperative to prevent the rapid progression of the disease. It remains unclear whether the Middle Eastern populations experience a more pronounced clinical progression of BD or if the issue lies in inadequate diagnosis and treatment.

Author Contributions

Conceptualization: Qusai Aljarrah, Mohamad Al-Badaineh, Mohammed Z. Allouh. **Data**

curation: Mohamad Al-Badaineh, Hammam Bani Salman, Mahmoud Megdadi. **Formal analysis:** Mahmoud Megdadi, Mohammed Z. Allouh. **Funding acquisition:** Mohammed Z. Allouh. **Investigation:** Qusai Aljarrah, Mohamad Al-Badaineh, Hammam Bani Salman, Nizar R. Alwaqfi, Mahmoud Megdadi, Mohammed Z. Allouh. **Methodology:** Mohamad Al-Badaineh, Hammam Bani Salman, Khalid A. Kheirallah, Mohammed Z. Allouh. **Project administration:** Qusai Aljarrah, Nizar R. Alwaqfi, Sohail Bakkar. **Resources:** Qusai Aljarrah, Nizar R. Alwaqfi, Sohail Bakkar, Mohammed Z. Allouh. **Validation:** Qusai Aljarrah, Sohail Bakkar, Khalid A. Kheirallah. **Supervision:** Qusai Aljarrah, Khalid A. Kheirallah, Mohammed Z. Allouh. **Writing – original draft:** Qusai Aljarrah, Mohamad Al-Badaineh, Mohammed Z. Allouh. **Writing – review & editing:** All authors. All authors have critically reviewed the manuscript, approved the final submitted version, and agreed to hold accountability for all aspects of the work.

Competing interests

The authors declare no competing interests exist.

Funding

This study was partially supported by a UPAR grant to M. Z. Allouh from the United Arab Emirates University (Grant code: G00004977, Fund no. 12M219).

Ethical consideration

This study was approved by the Institutional Review Board (IRB) committee at Jordan University of Science and Technology (JUST, approval Ref.: Feb2025/179-27), and it adheres to the ethical guidelines of the current version of the Declaration of Helsinki (2024). Due to the

retrospective nature of the study, the IRB committee at JUST waived the need for written patients' informed consent.

Availability of data

The dataset generated and analysed during the current study is available in the Figshare repository at: https://doi.org/10.6084/m9.figshare.30195802

References

1. Le Joncour A, Soudet S, Dupont A, Espitia O, Koskas F, Cluzel P, et al. Long-Term Outcome and Prognostic Factors of Complications in Thromboangiitis Obliterans (Buerger's Disease): A Multicenter Study of 224 Patients. J Am Heart Assoc. 2018;7: e010677. doi:10.1161/JAHA.118.010677

2. Puéchal X, Fiessinger J-N. Thromboangiitis obliterans or Buerger's disease: challenges for the rheumatologist. Rheumatology (Oxford). 2007;46: 192–199. doi:10.1093/rheumatology/kel388

3. Vijayakumar A, Tiwari R, Kumar Prabhuswamy V. Thromboangiitis Obliterans (Buerger's Disease)—Current Practices. Int J Inflam. 2013;2013: 156905. doi:10.1155/2013/156905

4. Olin JW. Thromboangiitis obliterans (Buerger's disease). N Engl J Med. 2000;343: 864–869. doi:10.1056/NEJM200009213431207

5. Lau H, Cheng SW. Buerger's disease in Hong Kong: a review of 89 cases. Aust N Z J Surg. 1997;67: 264–269. doi:10.1111/j.1445-2197.1997.tb01960.x

6. Fazeli B, Ligi D, Keramat S, Maniscalco R, Sharebiani H, Mannello F. Recent Updates and Advances in Winiwarter-Buerger Disease (Thromboangiitis Obliterans): Biomolecular Mechanisms, Diagnostics and Clinical Consequences. Diagnostics (Basel). 2021;11: 1736. doi:10.3390/diagnostics11101736

7. Klein-Weigel P, Volz TS, Zange L, Richter J. Buerger's disease: providing integrated care. J Multidiscip Healthc. 2016;9: 511–518. doi:10.2147/JMDH.S109985

8. Małecki R, Zdrojowy K, Adamiec R. Thromboangiitis obliterans in the 21st century--a new face of disease. Atherosclerosis. 2009;206: 328–334. doi:10.1016/j.atherosclerosis.2009.01.042

9. Cacione DG, Macedo CR, do Carmo Novaes F, Baptista-Silva JC. Pharmacological treatment for Buerger's disease. Cochrane Database Syst Rev. 2020;2020: CD011033. doi:10.1002/14651858.CD011033.pub4

10. Mills JL. Buerger's disease in the 21st century: diagnosis, clinical features, and therapy. Semin

Vasc Surg. 2003;16: 179–189. doi:10.1016/s0895-7967(03)00023-1

11. Lee SA, Jeong M-J, Ko G-Y, Hwang HS, Gwon DI, Choi E, et al. Long-term outcomes of large artery thromboangiitis obliterans and comparison with small artery thromboangiitis obliterans. Medicine (Baltimore). 2022;101: e28512. doi:10.1097/MD.0000000000028512

12. Olin JW, Young JR, Graor RA, Ruschhaupt WF, Bartholomew JR. The changing clinical spectrum of thromboangiitis obliterans (Buerger's disease). Circulation. 1990;82: IV3-8.

13. Sasaki S, Sakuma M, Yasuda K. Current status of thromboangiitis obliterans (Buerger's disease) in Japan. Int J Cardiol. 2000;75 Suppl 1: S175-181. doi:10.1016/s0167-5273(00)00190-x

14. Bae M, Chung SW, Lee J, Kim E, Kang G, Jin M. Early Diagnosis and Intervention Are Needed for a Reasonable Prognosis of Thromboangiitis Obliterans. J Chest Surg. 2023;56: 328–335. doi:10.5090/jcs.22.148

15. Bozkurt AK, Beşirli K, Köksal C, Sirin G, Yüceyar L, Tüzün H, et al. Surgical treatment of Buerger's disease. Vascular. 2004;12: 192–197. doi:10.1258/rsmvasc.12.3.192

16. Watanabe Y, Miyata T, Shigematsu K, Tanemoto K, Nakaoka Y, Harigai M, et al. Current Trends in Epidemiology and Clinical Features of Thromboangiitis Obliterans in Japan - A Nationwide Survey Using the Medical Support System Database. Circ J. 2020;84: 1786–1796. doi:10.1253/circj.CJ-19-1165

17. Papa MZ, Rabi I, Adar R. A point scoring system for the clinical diagnosis of Buerger's disease. Eur J Vasc Endovasc Surg. 1996;11: 335–339. doi:10.1016/s1078-5884(96)80081-5

18. Bura A, Auriol J, Rousseau H, Joffre F. Thromboangiitis Obliterans or Buerger's Disease. In: Hendaoui L, Stanson AW, Bouhaouala MH, Joffre F, editors. Systemic Vasculitis: Imaging Features. Berlin, Heidelberg: Springer; 2012. pp. 209–219. doi:10.1007/174_2011_151

19. Shionoya S. Diagnostic criteria of Buerger's disease. International Journal of Cardiology. 1998;66: S243–S245. doi:10.1016/S0167-5273(98)00175-2

20. Liu Z, Zhou C, Guo H, Wang M, Liang J, Zhang Y. Knowledge Mapping of Global Status and Trends for Thromboangiitis Obliterans: A Bibliometrics and Visual Analysis. J Pain Res. 2023;16: 4071–4087. doi:10.2147/JPR.S437521

21. al Zahrani HA. Arteritis: experience in Jeddah, Saudi Arabia. Cardiovasc Surg. 1993;1: 215–219.

22. Shionoya S. Diagnostic criteria of Buerger's disease. Int J Cardiol. 1998;66 Suppl 1: S243-245; discussion S247. doi:10.1016/s0167-5273(98)00175-2

23. Rutherford RB, Baker JD, Ernst C, Johnston KW, Porter JM, Ahn S, et al. Recommended standards for reports dealing with lower extremity ischemia: Revised version. Journal of Vascular Surgery. 1997;26: 517–538. doi:10.1016/S0741-5214(97)70045-4

24. Uyanık SA, Öğüşlü U, Aminu IS, Yılmaz B, Çevik H, Atlı E, et al. Endovascular Treatment of Critical Limb Ischemia in Buerger Disease (Thromboangiitis Obliterans) With Midterm Follow-Up: A Viable Option When Bypass Surgery Is Not Feasible. AJR Am J Roentgenol. 2021;216: 421–427. doi:10.2214/AJR.20.23023

25. Watanabe Y, Shimizu Y, Hashimoto T, Iwahashi T, Shigematsu K, Nakaoka Y, et al. Demographic Traits, Clinical Status, and Comorbidities of Patients With Thromboangiitis Obliterans in Japan. Circ J. 2024;88: 319–328. doi:10.1253/circj.CJ-23-0211

26. Choi B, Jang SY, Kim S-K, Kim N, Kim K, Kim DK. The incidence, prevalence, and survival rate of thromboangiitis obliterans in Korea: a retrospective population-based study. Cardiovasc Diagn Ther. 2020;10: 1238–1244. doi:10.21037/cdt-20-582

27. Fazeli B, Ravari H. A disease-specific activity score for Thromboangiitis obliterans. Vascular. 2014;22: 336–340. doi:10.1177/1708538113516315

28. De Haro J, Acin F, Bleda S, Varela C, Esparza L. Treatment of thromboangiitis obliterans (Buerger's disease) with bosentan. BMC Cardiovasc Disord. 2012;12: 5. doi:10.1186/1471-2261-12-5

29. Kim D-H, Ko Y-G, Ahn C-M, Shin D-H, Kim J-S, Kim B-K, et al. Immediate and late outcomes of endovascular therapy for lower extremity arteries in Buerger disease. J Vasc Surg. 2018;67: 1769–1777. doi:10.1016/j.jvs.2017.09.020

30. Fazeli B, Ravari H, Assadi R. Natural history definition and a suggested clinical approach to Buerger's disease: a case-control study with survival analysis. Vascular. 2012;20: 198–202. doi:10.1258/vasc.2011.oa0323

31. Ohta T, Ishioashi H, Hosaka M, Sugimoto I. Clinical and social consequences of buerger disease. Journal of Vascular Surgery. 2004;39: 176–180. doi:10.1016/j.jvs.2003.08.006

32. Soudet S, Le Joncour A, Quemeneur T, Maillard H, Koskas F, Hachulla E, et al. Did the Clinical Spectrum of Thromboangiitis Obliterans Change in the Past 40 Years? Angiology. 2020;71: 621–625. doi:10.1177/0003319720920163

33. Galyfos G, Liakopoulos D, Chamzin A, Sigala F, Filis K. A systematic review and meta-analysis of early and late outcomes after endovascular angioplasty among patients with thromboangiitis obliterans and chronic limb ischemia. J Vasc Surg. 2023;77: 1534-1541.e2. doi:10.1016/j.jvs.2022.09.017

34. Ghoneim BM, Karmota AG, Abuhadema AM, Shaker AA, Abdelmawla HM, Nasser MM, et al. Management of Buerger's Disease in Endovascular Era. Int J Angiol. 2019;28: 173–181. doi:10.1055/s-0039-1685200

35. Soliman M, Mowafy K, Elsaadany N, Soliman R, Elmetwally A. Thromboangiitis obliterans: Aggressive angioplasty provides a potential solution (randomized pilot study). SAGE Open Med. 2020;8: 2050312120927636. doi:10.1177/2050312120927636

36. Tavakoli H, Rezaii J, Esfandiari K, Salimi J, Rashidi A. Buerger's disease: a 10-year experience in Tehran, Iran. Clin Rheumatol. 2008;27: 369–371. doi:10.1007/s10067-007-0784-x

37. Olin JW. Thromboangiitis Obliterans: 110 Years Old and Little Progress Made. J Am Heart Assoc. 2018;7: e011214. doi:10.1161/JAHA.118.011214

38. Sasaki S, Sakuma M, Kunihara T, Yasuda K. Distribution of arterial involvement in thromboangiitis obliterans (Buerger's disease): results of a study conducted by the Intractable Vasculitis Syndromes

Research Group in Japan. Surg Today. 2000;30: 600–605. doi:10.1007/s005950070099

39. Fazeli B, Dadgar Moghadam M, Niroumand S. How to Treat a Patient with Thromboangiitis Obliterans: A Systematic Review. Ann Vasc Surg. 2018;49: 219–228. doi:10.1016/j.avsg.2017.10.022

40. Cooper LT, Tse TS, Mikhail MA, McBane RD, Stanson AW, Ballman KV. Long-term survival and amputation risk in thromboangiitis obliterans (Buerger's disease). J Am Coll Cardiol. 2004;44: 2410–2411. doi:10.1016/j.jacc.2004.09.029

41. Börner C, Heidrich H. Long-term follow-up of thromboangiitis obliterans. Vasa. 1998;27: 80–86.

42. Salimi J, Tavakkoli H, Salimzadeh A, Ghadimi H, Habibi G, Masoumi AA. Clinical characteristics of Buerger's disease in Iran. J Coll Physicians Surg Pak. 2008;18: 502–505.

43. Lee CY, Choi K, Kwon H, Ko G-Y, Han Y, Kwon T-W, et al. Outcomes of endovascular treatment versus bypass surgery for critical limb ischemia in patients with thromboangiitis obliterans. PLoS One. 2018;13: e0205305. doi:10.1371/journal.pone.0205305

44. Ye K, Shi H, Qin J, Yin M, Liu X, Li W, et al. Outcomes of endovascular recanalization versus autogenous venous bypass for thromboangiitis obliterans patients with critical limb ischemia due to tibioperoneal arterial occlusion. Journal of Vascular Surgery. 2017;66: 1133-1142.e1. doi:10.1016/j.jvs.2017.03.425

45. Ates A, Yekeler I, Ceviz M, Erkut B, Pac M, Basoglu A, et al. One of the most frequent vascular diseases in northeastern of Turkey: Thromboangiitis obliterans or Buerger's disease (experience with 344 cases). Int J Cardiol. 2006;111: 147–153. doi:10.1016/j.ijcard.2005.12.002

46. Kröger K, Kreuzfelder E, Moser C, Santosa F, Buss C, Grosse-Wilde H. Thrombangitis obliterans: leucocyte subpopulations and circulating immune complexes. Vasa. 2001;30: 189–194. doi:10.1024/0301-1526.30.3.189

47. Di X, Han W, Liu C-W, Ni L, Zhang R. A systematic review and meta-analysis on the association between C-reactive protein levels and adverse limb events after revascularization in patients with peripheral arterial disease. J Vasc Surg. 2021;74: 317–326. doi:10.1016/j.jvs.2021.02.026

48. Modaghegh M-HS, Hafezi S. Endovascular Treatment of Thromboangiitis Obliterans (Buerger's Disease). Vasc Endovascular Surg. 2018;52: 124–130. doi:10.1177/1538574417744085

49. Gundogmus CA, Samadli V, Sorkun M, Oguzkurt L. The Effect of Smoking Cessation on the Technical Success of Endovascular Treatment for Thromboangiitis Obliterans. J Vasc Interv Radiol. 2023;34: 1038–1044. doi:10.1016/j.jvir.2023.02.008

Figure Legends

Figure 1. Digital Subtraction Angiographic pattern of Buerger disease in a young male patient. (**A**) Occluded distal femoropopliteal segment with typical corkscrew collaterals and cutoff occlusions. (**B**) The distal tibial vessels of the same patient, with extensive attenuation of the plantar and digital arteries, with compensatory collateral circulation.

Figure 2. Sequential wound healing progression following amputation of the right great toe in a 27-year-old male diagnosed with Buerger disease, initially presenting with an RC V infected ischemic ulcer (**A**).

Table 1. General characteristics of patients with Buerger disease (thromboangiitis obliterans) at a referral center in Jordan.

Characteristic	Number	Percent (%)
	Mean ± SD	
**Sex**		
Male	39$^{\uparrow\uparrow}$	95.1
Female	2	4.9
**Mean Age at Diagnosis (y)**	38.4 ± 7.0	
**Age at Diagnosis (Category)**		
20 – 29 y	3	7.3
30 – 39 y	21$^{\uparrow\uparrow}$	51.2
40 – 49 y	16	39.0
50 – 59 y	1	2.4
**Mean Duration from Diagnosis (y)**	7.9 ± 6.8	
**Duration from Diagnosis**		
≤ 5 y	21	51.2
> 5 y	20	48.8
**Smoking Status**		
Current	33$^{\uparrow\uparrow}$	80.5
Former	8	19.5
Never	0	0
**Mean Smoking Duration (y)**	24.4 ± 10.5	
**Smoking Amount (pack/y)**		
< 20	4	9.8
≥ 20	37$^{\uparrow\uparrow}$	90.2
**Presence of Comorbidities (*diabetes, hypertension, and/or hyperlipidemia*)**		
Yes	12	29.3
No	29	70.7
**Laboratory Examination**		
High CRP (>10 mg/L)	24	75.0*
**Education**		
Primary school	9	22.0
Secondary school	21	51.2
Undergraduate degree	9	22.0
Postgraduate degree	2	4.9
**Job Loss**		
Yes	28$^{\uparrow}$	68.3
No	13	31.7
**Mean Hospital Readmission/y**	5.4 ± 5.8	
**Hospital Readmission/y**		
0 – 5	30$^{\uparrow\uparrow}$	73.2
6 – 10	5	12.2
11 – 15	3	7.3
> 15	3	7.3

Abbreviations: CRP, C-reactive protein; SD, standard deviation; y, year.
$^{\uparrow}$($p < 0.05$), $^{\uparrow\uparrow}$($p < 0.01$): significantly higher than expected frequency (χ^2 goodness of fit test). *Out of 32 patients since there were nine patients without CRP values in their medical records.

Table 2. Clinical presentations of patients with Buerger disease (thromboangiitis obliterans) at a referral center in Jordan.

	Number	Percent (%)
	Mean ± SD	
**Number of Limbs Affected**		
1	20	48.8
2	20	48.8
3	1	2.4
**Rutherford Classification**		
Category 4 (rest pain)	3↓↓	7.3
Category 5 (ulcer)	25↑↑	61.0
Category 6 (gangrene)	13	31.7
**Presence of Ischemic Ulcer**		
Unilateral lower limb	18↑↑	43.9
Bilateral lower limb	14	34.1
Upper and unilateral lower limbs	4	9.8
Upper and Bilateral lower limbs	1	2.4
Upper limb alone	1	2.4
None	3	7.3
**Involved Artery**		
Anterior tibial artery	5	12.2
Posterior tibial artery	4	9.8
Both	32↑↑	78.0
**Multilevel Disease**		
Yes	19	46.3
No	22	53.7
**Thrombophlebitis**		
Yes	14	34.1
No	27	65.9
**Infection at Admission**		
Yes	13	31.7
No	28	68.3

Abbreviations: SD, standard deviation.
↑↑($p < 0.01$): significantly higher than expected frequency (χ^2 goodness of fit test).
↓↓($p < 0.01$): significantly lower than expected frequency (χ^2 goodness of fit test).

Table 3. Treatment methods and outcomes of patients with Buerger disease (thromboangiitis obliterans) at a referral center in Jordan.

	Number	Percent (%)
	Mean ± SD	
**Treatment Method**		
Medical therapy alone	8	19.5
Revascularization	33	80.5
**Revascularization Procedure**		
Endovascular procedure	19$^{\uparrow}$	46.3
Surgical procedure	5	12.2
Both	9	22.0
**Patient Outcome**		
No amputation	15	36.6
Minor amputation	15	36.6
Major amputation	11	26.8
**Amputation Location**		
Lower limb	24$^{\uparrow\uparrow}$	58.5
Upper limb	1	2.4
Both	1	2.4
No amputations	15	36.6

$^{\uparrow}$($p < 0.05$), $^{\uparrow\uparrow}$($p < 0.01$): significantly higher than expected frequency (χ^2 goodness of fit test).

Table 4. General characteristic factors associated with the outcomes of patients with Buerger disease (thromboangiitis obliterans) at a referral center in Jordan.

Characteristic	No amputation	Minor amputation	Major amputation	p-value*
**Sex**				
Male	14 (35.9)	14 (35.9)	11 (28.2)	0.680
Female	1 (50.0)	1 (50.0)	0 (0.0)	
**Mean Age at Diagnosis (y)**	39.7 ± 7.4	37.2 ± 6.1	38.4 ± 7.9	0.637
**Categorical Age at Diagnosis**				
20 – 29 y	1 (33.3)	1 (33.3)	1 (33.3)	
30 – 39 y	7 (33.3)	9 (42.9)	5 (23.8)	0.879
40 – 49 y	6 (37.5)	5 (31.2)	5 (31.2)	
50 – 59 y	1 (100.0)	0 (0.0)	0 (0.0)	
**Mean Duration from diagnosis (y)**	5.9 ± 5.5	6.8 ± 5.9	12.1 ± 8.1$^{\uparrow}$	0.048
**Duration from Diagnosis**				
≤ 5 y	10 (52.6)	8 (42.1)	1 (5.3)$^{\downarrow}$	0.012
> 5 y	5 (22.7)	7 (31.8)	10 (45.5)$^{\uparrow}$	
**Smoking Status**				
Current	13 (39.4)	10 (30.3)	10 (30.3)	0.229
Former	2 (25.0)	5 (62.5)	1 (12.5)	
**Smoking Duration**				
≤ 20 y	5 (27.8)	10 (55.6)	3 (16.7)	0.079
> 20 y	10 (43.5)	5 (21.7)	8 (34.8)	
**Presence of Comorbidities**				
Yes	5 (41.7)	3 (25.0)	4 (33.3)	0.604
No	10 (34.5)	12 (41.4)	7 (24.1)	
**Education**				
Primary school	3 (33.3)	2 (22.2)	4 (44.4)	
Secondary school	8 (38.1)	8 (38.1)	5 (23.8)	0.495
Undergraduate degree	4 (44.4)	3 33.3)	2 (22.2)	
Postgraduate degree	0 (0.0)	2 (100.0)	0 (0.0)	
**Family History**				
Yes	0 (0.0)	2 (50.0)	2 (50.0)	0.256
No	15 (40.5)	13 (35.1)	9 (24.3)	
**Job Loss**				
Yes	2 (7.1)	15 (53.6)$^{\uparrow\uparrow}$	11 (39.3)$^{\uparrow\uparrow}$	< 0.001
No	13 (100.0)$^{\uparrow\uparrow}$	0 (0.0)	0 (0.0)	
**Hospital Readmission**				
Up to 3 per year	11 (50.0)$^{\uparrow}$	9 (40.9)	2 (9.1)	0.017
More than 3 per year	4 (21.1)	6 (31.6)	9 (47.4)$^{\uparrow}$	
**Diagnosis Delay**				
< 1 y	6 (42.9)	4 (28.6)	4 (28.6)	0.731
≥ 1 y	9 (33.3)	11 (40.7)	7 (25.9)	

Abbreviations: p, probability; y, year. Data are presented as mean ± standard deviation for continuous values and as frequency (%) for categorical values. $^{\uparrow}$($p < 0.05$), $^{\uparrow\uparrow}$($p < 0.01$): significantly higher than other groups. $^{\downarrow}$($p < 0.05$): significantly lower than other groups. *p-value was calculated using Pearson's chi-squared test for categorical variables and one-way ANOVA for continuous variables.

Table 5. Clinical factors associated with the outcomes of patients with Buerger disease (thromboangiitis obliterans) at a referral center in Jordan.

Characteristic	No amputation	Minor amputation	Major amputation	p-value*
**Mean CRP value (mg/L)**	10.7 ± 8.7$^{\downarrow}$	36.0 ± 24.1	43.6 ± 43.3	0.025
**CRP Value**				0.041
Normal CRP (≤ 10 mg/L)	6 (75.0)$^{\uparrow}$	1 (12.5)	1 (12.5)	
High CRP (> 10 mg/L)	6 (25.0)	9 (37.5)	9 (37.5)	
**Number of Affected Limbs**				0.136
1	7 (35.0)	10 (50.0)	3 (15.0)	
2 or more	8 (38.1)	5 (23.8)	8 (38.1)	
**Rutherford Classification**				< 0.001
Category 4 (rest pain)	3 (100.0)$^{\uparrow}$	0 (0.0)	0 (0.0)	
Category 5 (ulcer)	11 (44.0)	12 (48.0)	2 (8.0)	
Category 6 (gangrene)	1 (7.7)	3 (23.1)	9 (69.2)$^{\uparrow\uparrow}$	
**Presence of Ischemic Ulcer on Lower Limb**				0.420
Unilateral lower limb	7 (31.8)	10 (45.5)	5 (22.7)	
Bilateral lower limb	5 (33.3)	4 (26.7)	6 (40.0)	
**Involved arteries**				0.102
Single (ATA or PTA)	6 (66.7)	2 (22.2)	1 (11.1)	
Both (ATA & PTA)	9 (28.1)	13 (40.6)	10 (31.2)	
**Multilevel Disease**				0.405
Yes	6 (31.6)	9 (47.4)	4 (21.1)	
No	9 (40.9)	6 (27.3)	7 (31.8)	
**Thrombophlebitis**				0.793
Yes	6 (42.9)	5 (35.7)	3 (21.4)	
No	9 (33.3)	10 (37.0)	8 (29.6)	
**Infection at Admission**				0.001
Yes	1 (7.7)	4 (30.8)	8 (61.5)$^{\uparrow\uparrow}$	
No	14 (50.0)$^{\uparrow}$	11 (39.3)	3 (10.7)	
**Treatment Method**				0.674
Medical therapy alone	3 (37.5)	2 (25.0)	3 (37.5)	
Revascularization	12 (36.4)	13 (39.4)	8 (24.2)	

Abbreviations: ATA, anterior tibial artery; PTA, posterior tibial artery; p, probability. Data are presented as frequency (%). $^{\uparrow}$($p < 0.05$), $^{\uparrow\uparrow}$($p < 0.01$): significantly higher than other groups. $^{\downarrow}$($p < 0.05$): significantly lower than other groups. *p-value was calculated using Pearson's chi-squared test.

Table 6. Multivariable ordinal logistic regression model for the prediction of major amputation in patients with Buerger disease (thromboangiitis obliterans) at a referral center in Jordan.

The Predictors	P-value	Odds Ratio	95% CI	
			Lower	Upper
**CRP level**	0.035	1.044	1.003	1.087
**Duration of the Disease**				
> 5 y	0.140	-	0.631	25.795
≤ 5 y	Ref	Ref	Ref	Ref
**Rutherford Class***				
Category 6 (gangrene)	0.021	11.545	1.444	92.332
Category 5 (ulcer)	Ref	Ref	Ref	Ref
**Infection at Admission**				
Yes	0.033	7.745	1.182	50.755
No	Ref	Ref	Ref	Ref

Abbreviations: P, probability; CI, confidence interval. The logistic regression model included the four variables reported in this table. *Rutherford category 4 (rest pain) was excluded from the regression model since it only included 3 patients.

Figure 1.DSA Click here to access/download;Figure;Figure 1. DSA.jpeg

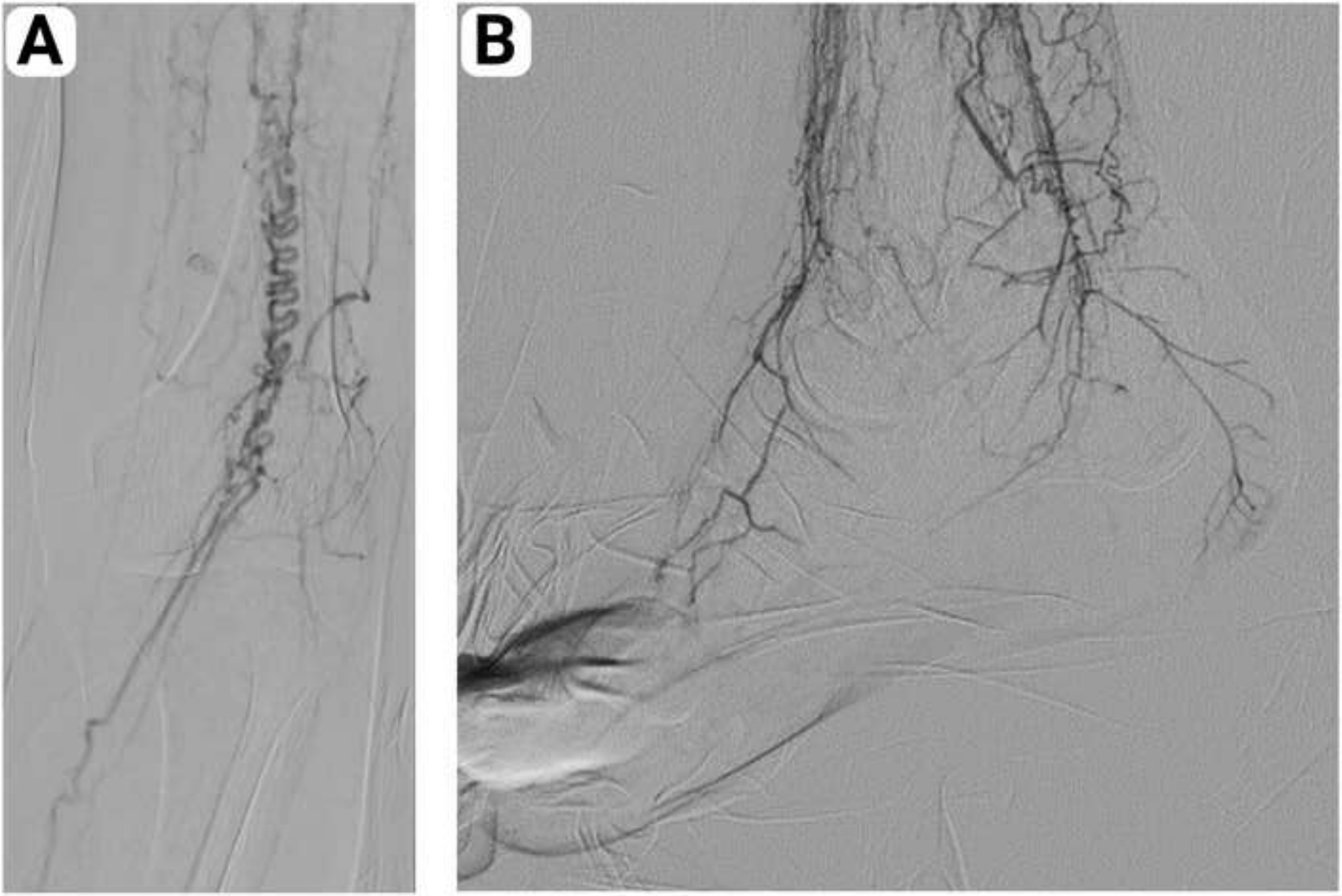

Figure 2. BD, healing after amputation

Click here to access/download;Figure;Figure 2. BD_healing after amputation.jpeg

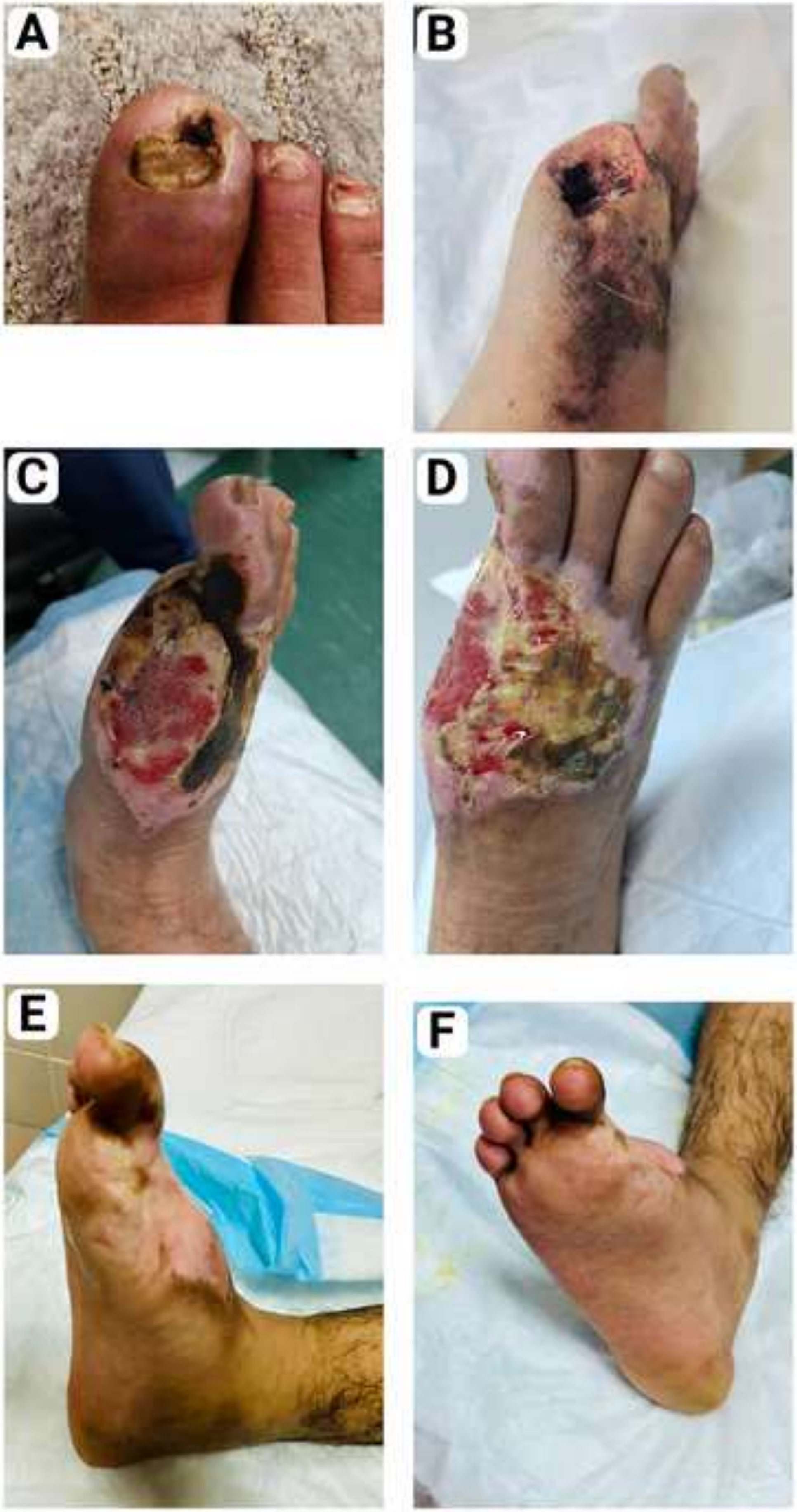

Supplement: Attachment — Submitted filename: PONE-D-25-59692_reviewer CommentsPedroDiogenes.pdf [file pone.0351071.s001.pdf]
