# Supplementary material for: Clinical outcomes of critical limb ischemia in Buerger disease: A contemporary analysis from an academic referral center in Jordan
Source: PLoS One. 2026 Jun 10;21(6):e0351071. doi: 10.1371/journal.pone.0351071 (PMC13252764; doi:10.1371/journal.pone.0351071)
Supplement: Attachment — Submitted filename: Response to Reviewers.pdf [file pone.0351071.s002.pdf]

Response Letter

PLOS One

Manuscript ID: PONE-D-25-59692

Manuscript Title: Clinical Outcomes of Critical Limb Ischemia in Buerger Disease: A Contemporary Analysis from an Academic Referral Center in Jordan

Dear Editor and Reviewers,

We sincerely thank you for your valuable and constructive comments provided to improve this manuscript. Changes have been made in the manuscript according to the comments and suggestions. All corrections in the revised manuscript are marked by the track changes option in the copy labeled "Revised Manuscript with Track Changes". Additionally, an unmarked version of the revised manuscript is provided in the file labeled "Manuscript".

Finally, a detailed point-by-point response to the comments is provided herewith. Please note that your comments are in black text, and our responses are in blue.

Journal Requirements

1. Please ensure that your manuscript meets PLOS ONE's style requirements, including those for file naming. The PLOS ONE style templates can be found at https://journals.plos.org/plosone/s/file?id=wjVg/PLOSOne_formatting_sample_main_body.pdf and https://journals.plos.org/plosone/s/file?id=ba62/PLOSOne_formatting_sample_title_authors_affiliations.pdf

RESPONSE: Done. The manuscript has been thoroughly revised to meet PLOS ONE style requirements.

2. Your ethics statement should only appear in the Methods section of your manuscript. If your ethics statement is written in any section besides the Methods, please delete it from any other section.

RESPONSE: Done. The ethical consideration statement has been included in the Methods section only. **[page 4, lines 86–91 in the unmarked version]**

3. Thank you for stating the following financial disclosure:

This study was partially supported by a UPAR grant to M. Z. Allouh from the United Arab Emirates University (Grant code: G00004977, Fund no. 12M219).

Please state what role the funders took in the study. If the funders had no role, please state: "The funders had no role in study design, data collection and analysis, decision to publish, or preparation of the manuscript."

If this statement is not correct you must amend it as needed.

Please include this amended Role of Funder statement in your cover letter; we will change the online submission form on your behalf.

RESPONSE: Done. The funder had no role in the study, and the statement recommended above has now been included in the “Funding” section of the manuscript.

[page 23, lines 444–445 in the unmarked version]

4. We note that Figure(s) 1, 2 in your submission contain copyrighted images. All PLOS content is published under the Creative Commons Attribution License (CC BY 4.0), which means that the manuscript, images, and Supporting Information files will be freely available online, and any third party is permitted to access, download, copy, distribute, and use these materials in any way, even commercially, with proper attribution. For more information, see our copyright guidelines: http://journals.plos.org/plosone/s/licenses-and-copyright.

We require you to either (1) present written permission from the copyright holder to publish these figures specifically under the CC BY 4.0 license, or (2) remove the figures from your submission:

a. You may seek permission from the original copyright holder of Figure(s) 1, 2 to publish the content specifically under the CC BY 4.0 license.

We recommend that you contact the original copyright holder with the Content Permission Form (http://journals.plos.org/plosone/s/file?id=7c09/content-permission-form.pdf) and the following text:

"I request permission for the open-access journal PLOS ONE to publish XXX under the Creative Commons Attribution License (CCAL) CC BY 4.0 (http://creativecommons.org/licenses/by/4.0/). Please be aware that this license allows unrestricted use and distribution, even commercially, by third parties. Please reply and provide explicit written permission to publish XXX under a CC BY license and complete the attached form."

Please upload the completed Content Permission Form or other proof of granted permissions as an "Other" file with your submission.

In the figure caption of the copyrighted figure, please include the following text: "Reprinted from [ref] under a CC BY license, with permission from [name of publisher], original copyright [original copyright year]."

b. If you are unable to obtain permission from the original copyright holder to publish these figures under the CC BY 4.0 license or if the copyright holder's requirements are incompatible with the CC BY 4.0 license, please either i) remove the figure or ii) supply a replacement figure that complies with the CC BY 4.0 license. Please check copyright information on all replacement figures and update the figure caption with source information. If applicable, please specify in the figure caption text when a figure is similar but not identical to the original image and is therefore for illustrative purposes only.

RESPONSE: Thank you for this important comment. We would like to clarify that the images included in Figures 1 and 2 are **original patient images obtained by our team** and are not reproduced from any previously published work. To fully address the journal's

copyright and licensing requirements, we have replaced the previous versions of these figures with newly prepared figures constructed in PowerPoint using only our original images and author-generated labels and layout elements. Accordingly, the revised Figures 1 and 2 **do not** contain third-party copyrighted material and are fully compliant with the journal's CC BY 4.0 licensing requirements.

5. If the reviewer comments include a recommendation to cite specific previously published works, please review and evaluate these publications to determine whether they are relevant and should be cited. There is no requirement to cite these works unless the editor has indicated otherwise.

RESPONSE: There were no reviewer recommendations to cite any specific previously published works.

6. Please review your reference list to ensure that it is complete and correct. If you have cited papers that have been retracted, please include the rationale for doing so in the manuscript text, or remove these references and replace them with relevant current references. Any changes to the reference list should be mentioned in the rebuttal letter that accompanies your revised manuscript. If you need to cite a retracted article, indicate the article's retracted status in the References list and also include a citation and full reference for the retraction notice.

RESPONSE: The reference list was prepared using Zotero software in accordance with the PLOS One guidelines. No retracted papers were cited in the reference list.

Reviewer #1

1) Page 7: Access to full data is not available by clicking the link (403 Forbidden)

RESPONSE:

Thank you for this important technical comment. We have checked access to the data, and the link is functioning properly. Screenshots are embedded below as evidence. However, access may be denied in some cases, most likely due to technical difficulties or restrictions related to geolocation or internet service providers. Therefore, in case of difficulty accessing the data, researchers may contact the corresponding author to obtain the data file. The following statement has been added to the “Availability of data” section: The original dataset is also available from the corresponding author in case of difficulty accessing the link.

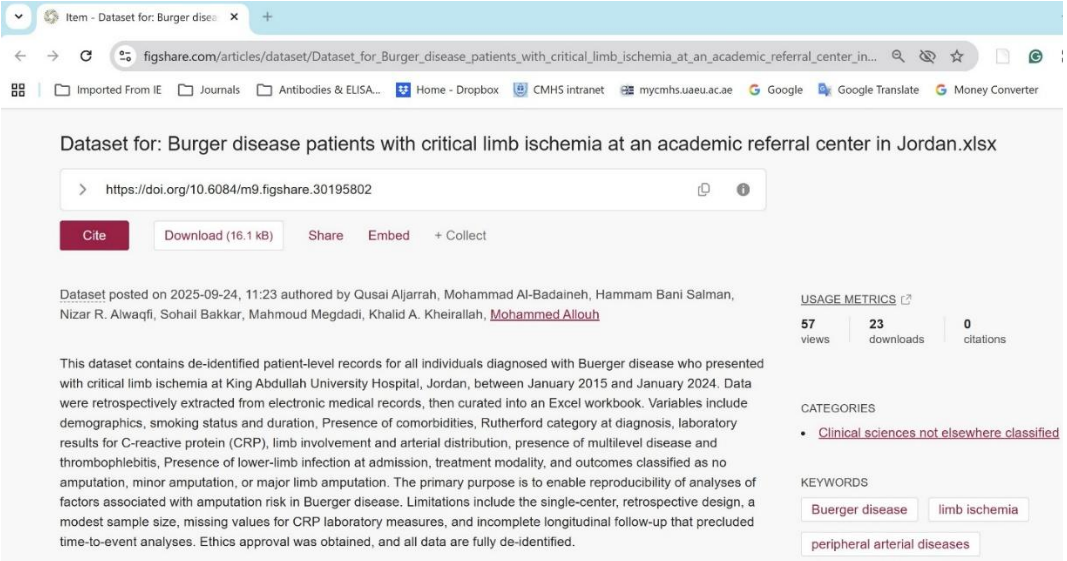
Item - Dataset for: Burger disea

figshare.com/articles/dataset/Dataset_for_Burger_disease_patients_with_critical_limb_ischemia_at_an_academic_referral_center_in...

Imported From IE | Journals | Antibodies & ELISA... | Home - Dropbox | CMHS intranet | mycmhs.uaeu.ac.ae | Google | Google Translate | Money Converter

Dataset for: Burger disease patients with critical limb ischemia at an academic referral center in Jordan.xlsx

https://doi.org/10.6084/m9.figshare.30195802

Cite | Download (16.1 kB) | Share | Embed | + Collect

Dataset posted on 2025-09-24, 11:23 authored by Qusai Aljarrah, Mohammad Al-Badaineh, Hammam Bani Salman, Nizar R. Alwaqfi, Sohail Bakkar, Mahmoud Megdadi, Khalid A. Kheirallah, Mohammed Allouh

This dataset contains de-identified patient-level records for all individuals diagnosed with Buerger disease who presented with critical limb ischemia at King Abdullah University Hospital, Jordan, between January 2015 and January 2024. Data were retrospectively extracted from electronic medical records, then curated into an Excel workbook. Variables include demographics, smoking status and duration, Presence of comorbidities, Rutherford category at diagnosis, laboratory results for C-reactive protein (CRP), limb involvement and arterial distribution, presence of multilevel disease and thrombophlebitis, Presence of lower-limb infection at admission, treatment modality, and outcomes classified as no amputation, minor amputation, or major limb amputation. The primary purpose is to enable reproducibility of analyses of factors associated with amputation risk in Buerger disease. Limitations include the single-center, retrospective design, a modest sample size, missing values for CRP laboratory measures, and incomplete longitudinal follow-up that precluded time-to-event analyses. Ethics approval was obtained, and all data are fully de-identified.

USAGE METRICS
57 views | 23 downloads | 0 citations

CATEGORIES
- Clinical sciences not elsewhere classified

KEYWORDS
Buerger disease | limb ischemia | peripheral arterial diseases

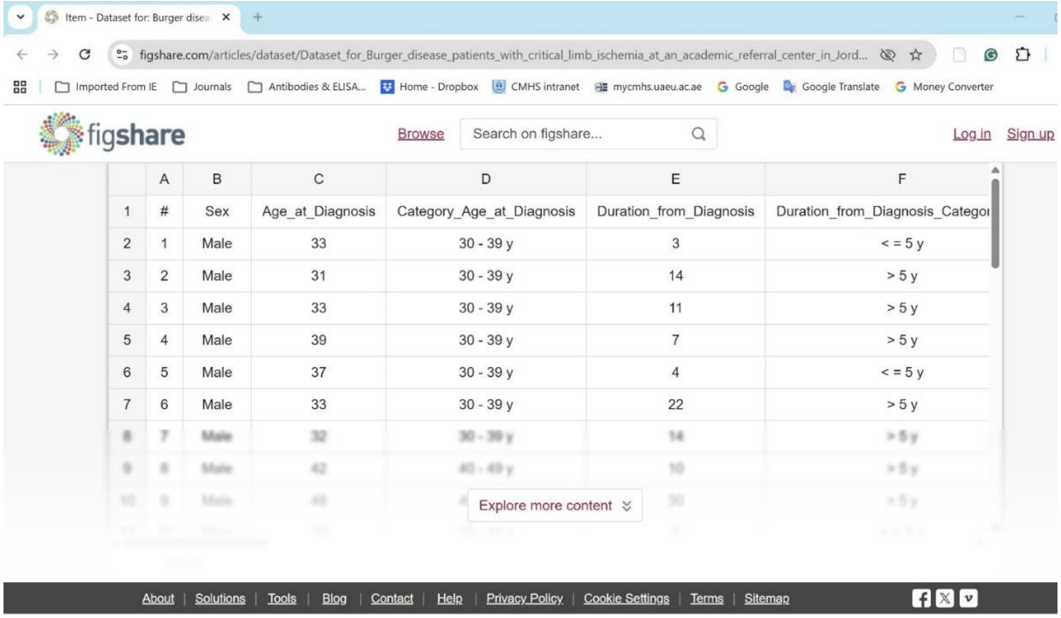
Item - Dataset for: Burger disea

figshare.com/articles/dataset/Dataset_for_Burger_disease_patients_with_critical_limb_ischemia_at_an_academic_referral_center_in_Jord...

Imported From IE | Journals | Antibodies & ELISA... | Home - Dropbox | CMHS intranet | mycmhs.uaeu.ac.ae | Google | Google Translate | Money Converter

figshare | Browse | Search on figshare... | Log in | Sign up

	A	B	C	D	E	F
1	#	Sex	Age_at_Diagnosis	Category_Age_at_Diagnosis	Duration_from_Diagnosis	Duration_from_Diagnosis_Catego
2	1	Male	33	30 - 39 y	3	< = 5 y
3	2	Male	31	30 - 39 y	14	> 5 y
4	3	Male	33	30 - 39 y	11	> 5 y
5	4	Male	39	30 - 39 y	7	> 5 y
6	5	Male	37	30 - 39 y	4	< = 5 y
7	6	Male	33	30 - 39 y	22	> 5 y

Explore more content

About | Solutions | Tools | Blog | Contact | Help | Privacy Policy | Cookie Settings | Terms | Sitemap

2) Page 14: Lines 120-121. In theory, the clinical diagnosis of Buerger's Disease required the presence of Shionoya's 5 criteria, until the emergence of the VAS consensus criteria in 2023, which began to associate angiographic and histopathological findings.

RESPONSE:

Thank you for this insightful comment, which raises an important point. We agree that the diagnosis of Buerger disease has evolved, and that the 2023 VAS Delphi consensus provides a more robust contemporary framework by incorporating angiographic and histopathological findings.

However, our study is a retrospective analysis of patients diagnosed **between January 2015 and January 2024**, and thus, the great majority of cases were identified **before** the publication of these updated criteria in October 2023. For this reason, our diagnostic approach necessarily reflects the standards and **real-world vascular practice that prevailed during the study period**.

In our setting, Shionoya's criteria, together with characteristic angiographic findings, represented the practical and clinically accepted framework used to establish the diagnosis. This approach also allows valid comparison with the existing literature, since many previously published studies on Buerger disease have used similar criteria. Importantly, we did not rely on Shionoya's criteria in isolation, but combined them with angiographic evidence and radiological exclusion of atherosclerotic disease to improve diagnostic specificity and minimize misclassification.

3) Page 14: Line 122. Did the patients undergo tissue biopsy of the blood vessels?

RESPONSE:

Thank you again for raising this point. As explained above, the assessment of the patients did not involve tissue biopsy of the blood vessels. Instead, their evaluation was based on radiological imaging techniques to determine the absence of atherosclerotic plaques. The term "radiological absence" has been included into the revised manuscript. **[page 5, line 100 in the unmarked version]**

4) Page 15: Lines 144-145. The simple advice to quit smoking has low effectiveness in the literature if it is not associated with follow-up in support groups and/or the use of pharmacological support. This may be one of the factors associated with the high amputation rate found in the cohort and should be discussed.

RESPONSE:

We fully agree with this important and clinically relevant observation. Indeed, simple advice to stop smoking, when provided without structured follow-up, behavioral support, or pharmacological treatment, has limited effectiveness according to the literature. We agree that this may have contributed to the high amputation rate observed in our cohort and therefore merits explicit discussion in the manuscript.

This issue is particularly relevant in our setting. In Jordan, smoking cessation services remain limited, with a lack of well-established smoking cessation clinics and structured cessation programs. In addition, smoking cessation medications are generally not covered by insurance, which further restricts patients' access to effective therapeutic support. As a result, many patients are left with advice alone, without the multidisciplinary follow-up and treatment strategies needed to address the physiological and behavioral components of nicotine dependence.

To address your valuable comment, we have now added the following paragraph to the Discussion section:

"It is important to emphasize that simple advice to quit smoking, when not accompanied by structured follow-up, behavioral support, and pharmacological treatment, has only limited effectiveness in the literature [48,49]. This may represent one of the factors contributing to the high amputation rate observed in our cohort. Effective smoking cessation usually requires structured programs that incorporate both pharmacological aids and behavioral interventions targeting the physiological and psychological aspects of nicotine dependence. Unfortunately, the healthcare system in Jordan lacks robust smoking cessation clinics, and smoking cessation medications are not generally covered

by insurance, which limits patients' access to the support and treatment necessary for successful cessation." **[pages 19–20, lines 366–374 in the unmarked version]**

New References:

48. Stead LF, Buitrago D, Preciado N, Sanchez G, Hartmann-Boyce J, Lancaster T. Physician advice for smoking cessation. Cochrane Database Syst Rev. 2013;2013: CD000165. doi:10.1002/14651858.CD000165.pub4

49. Stead LF, Koilpillai P, Fanshawe TR, Lancaster T. Combined pharmacotherapy and behavioural interventions for smoking cessation. Cochrane Database Syst Rev. 2016;3: CD008286. doi:10.1002/14651858.CD008286.pub3

5) Page 16: Line 176. In the results, I observe a lack of reference to how many cases were retrospectively identified, the mechanism used to search for the cases, and, among the identified cases, how many presented complete data and in which aspects they were incomplete. How many were lost to follow-up?

RESPONSE:

We agree that the case identification process, data completeness, and follow-up availability should be described more explicitly for clarity and transparency.

In response, we have clarified in the manuscript that this retrospective study included **41 retrospectively identified eligible patients** with Buerger disease and critical limb ischemia who were extracted from the electronic medical records of King Abdullah University Hospital during the study period from January 2015 to January 2024. Case identification was performed by **first** searching the hospital electronic medical records using the keyword "**critical limb ischemia**" and **then further** identifying patients with Buerger disease using the keyword "**Buerger disease**." The **diagnosis was then verified** based on the predefined study diagnostic criteria.

We have also clarified data completeness. Of the 41 included patients, **9 patients had missing CRP values**, whereas the remaining baseline study variables were complete for the rest of the cohort. This point has now been stated explicitly in the revised manuscript.

Regarding follow-up, we agree that this is an important issue. However, because follow-up in this retrospective cohort was irregular and many patients had intermittent consultations, the exact number of patients lost to follow-up **could not be determined reliably from the available records**. For this reason, we did not perform longitudinal follow-up analyses, and we have clarified this more explicitly in the manuscript **as a limitation**.

To improve transparency, these methodological details and the extent of missing data have now been added to the revised manuscript as follows:

Methods section – Study protocol – first sentence:

Cases were identified retrospectively from the electronic medical records of King Abdullah University Hospital affiliated with JUST between January 2015 and January 2024 by searching the keyword "critical limb ischemia" and then identifying patients with BD using the keyword "Buerger disease." **[page 5, lines 94–97 in the unmarked version]**

Results section – General and clinical characteristics – first and second sentences:

A total of 41 eligible patients were retrospectively identified and included. Missing baseline data were limited to CRP values in 9 patients; all other baseline variables were complete.

[page 7, lines 158–160 in the unmarked version]

Discussion section – Limitations paragraph – sentence in bold text:

Additionally, follow-up data were often unavailable due to intermittent consultations, with gaps frequently attributable to patients' low socioeconomic status and health insurance limitations. **Therefore, the exact number of patients lost to follow-up could not be determined reliably from the available records.** These incomplete and irregular data precluded the application of time-to-event analyses, such as Kaplan–Meier curves for amputation-free survival.

[page 21, lines 396–397 in the unmarked version]

6) Page 24: Lines 347-348. This goes against the common notion that smoking cessation is a key factor in the treatment of Buerger's disease." Smoking cessation not only halts

disease progression but has shown to effectively reduce the risk of major limb amputations." https://doi.org/10.1016/j.jss.2024.12.034

RESPONSE:

We apologize if our original wording may have created an unintended impression. We fully agree that smoking cessation is the cornerstone of treatment in Buerger's disease and is associated with lower rates of vascular events and amputation. This is supported by more recent evidence, including a multicenter study showing that patients who stopped tobacco had a significantly lower risk of amputation than those who continued, and by a recent systematic review emphasizing smoking cessation as a key measure to reduce the risk of major limb amputation.

Our intention was not to suggest that smoking cessation is ineffective or unimportant. Rather, our point was that, in patients who already present with advanced limb-threatening ischemia, smoking cessation may not immediately abolish the risk of limb loss because **irreversible vascular and tissue damage may already be present** at the time of presentation. This interpretation is consistent with our cohort, in which 93% of patients presented with advanced critical limb ischemia, Rutherford category V or VI, and with our smoking analysis, where former smokers had a numerically lower rate of major limb amputation than current smokers, 12.5% versus 30.3%, although this difference did not reach statistical significance.

In addition, smoking status in our retrospective cohort was based on self-report and was not biochemically verified, which may have reduced the accuracy of the smoking-related analysis and attenuated the observed association.

To avoid any misunderstanding, we have revised the Discussion part by **removing the sentence** that could be interpreted as minimizing the value of smoking cessation, and we have replaced it with a **more balanced statement** emphasizing that smoking cessation remains essential in Buerger's disease, although its protective effect may be less immediately evident in patients who present with advanced irreversible ischemic damage.

Here are the newly revised sentences:

Nevertheless, smoking cessation remains the cornerstone of management in BD and is associated with a lower risk of vascular events and amputation. However, in patients who present with advanced critical limb ischemia and established tissue loss, cessation may not immediately eliminate the risk of limb loss because irreversible vascular and tissue damage may already be present.

[page 19, lines 362–365 in the unmarked version]

Reviewer #2

1. In this study, 33 (80.5%) patients had revascularization for the treatment of Buerger's disease (Table 3). Please provide information on the patency rate of revascularization and changes in ischemic symptoms (ulcer, gangrene) during follow-up. This information helps us to understand the effectiveness of revascularization for Buerger's disease.

RESPONSE:

Thank you for this clinically relevant comment. We fully agree that post-revascularization patency and interval changes in ischemic manifestations, such as ulcer healing and gangrene progression or regression, would provide valuable insight into the effectiveness of revascularization in Buerger disease.

However, due to the retrospective design of this study, these outcomes could not be assessed to support a reliable formal analysis. The post-procedural radiological surveillance was not performed according to a standardized institutional protocol, and routine surveillance duplex examinations were not consistently available in our vascular practice during the study period. In addition, follow-up attendance was irregular, and serial documentation of ischemic wound status, including ulcer healing and gangrene evolution, was not uniformly recorded in the medical records. As a result, any attempt to calculate patency rates or longitudinal symptom changes would have been based on incomplete and non-systematic data and could therefore introduce substantial bias.

For this reason, we elected not to present post-revascularization patency or symptom-evolution analyses, as we believe that doing so from incomplete retrospective follow-up data would risk overstating the strength of the evidence. Instead, we focused on the more consistently documented clinical endpoints available across the cohort, particularly limb amputation outcomes. In the revised manuscript, we have clarified this limitation more explicitly. We have now added explicit clarification in the Discussion that the absence of standardized radiological surveillance and the irregularity of follow-up prevented reliable assessment of revascularization patency and interval changes in ischemic lesions.

The following sentence has been added to the limitations paragraph:

Also, Post-revascularization patency rates and interval changes in ischemic lesions, including ulcer healing and gangrene progression or regression, could not be analyzed reliably because routine surveillance duplex imaging was not performed according to a standardized protocol, and follow-up documentation was incomplete and irregular as explained earlier.

[page 21, lines 399–403 in the unmarked version]

2. There were no significant relationships between smoking status at enrollment and the risk of amputation (Table 4). Smoking cessation is important for preventing disease progression in Buerger's disease. Please provide information about the effects of smoking cessation on outcomes.

RESPONSE:

Thank you for this important comment. We completely agree that the role of smoking cessation in managing Buerger disease cannot be overstated. The following two paragraphs have been included to the Discussion section of the revised manuscript regarding information on the effects of smoking cessation in Burger's disease outcomes:

"Our findings indicate that smoking status and duration did not influence limb salvage rates among the studied cohort. Former smokers were less likely to undergo MLA (12.5%) than current smokers (30.3%), although this difference was not statistically

significant (p= 0.229). A similar trend was observed in relation to smoking duration; patients who smoked > 20 years had a 34.8% incidence of MLA, while only 16.7% underwent MLA in patients who smoked ≤20 years (p=0.079). There are several explanations for this. **First**, smoking status was self-reported without urine cotinine verification, potentially leading to false claims of cessation. **Second**, our patients often ceased smoking only when confronted with severe disease progression. **Third**, some individuals consider non-cigarette tobacco use as cessation, and Jordan lacks smoking cessation education and resources. **Fourth**, the studied cohort presented with adverse limb events that possibly rendered smoking cessation insufficient to prevent limb loss. **Nevertheless, smoking cessation remains the cornerstone of management in BD and is associated with a lower risk of vascular events and amputation. However, in patients who present with advanced critical limb ischemia and established tissue loss, cessation may not immediately eliminate the risk of limb loss because irreversible vascular and tissue damage may already be present.**

It is important to emphasize that simple advice to quit smoking, when not accompanied by structured follow-up, behavioral support, and pharmacological treatment, has only limited effectiveness in the literature [48,49]. This may represent one of the factors contributing to the high amputation rate observed in our cohort. Effective smoking cessation usually requires structured programs that incorporate both pharmacological aids and behavioral interventions targeting the physiological and psychological aspects of nicotine dependence. Unfortunately, the healthcare system in Jordan lacks robust smoking cessation clinics, and smoking cessation medications are not generally covered by insurance, which limits patients' access to the support and treatment necessary for successful cessation."

New References:

48. Stead LF, Buitrago D, Preciado N, Sanchez G, Hartmann-Boyce J, Lancaster T. Physician advice for smoking cessation. Cochrane Database Syst Rev. 2013;2013: CD000165. doi:10.1002/14651858.CD000165.pub4

49. Stead LF, Koilpillai P, Fanshawe TR, Lancaster T. Combined pharmacotherapy and behavioural interventions for smoking cessation. Cochrane Database Syst Rev. 2016;3: CD008286. doi:10.1002/14651858.CD008286.pub3

[pages 19–20, lines 351–374 in the unmarked version]

Additionally, you may refer to Reviewer 1 comments number 4 and 6 about the role of smoking cessation in the management of Buerger disease.

3. What is the definition of "family history"? (Table 4)

RESPONSE: Thank you for drawing our attention to this information. Family history refers to **first degree relatives**, specifically denotes parents, siblings, and offspring. This information has now been included to the footnote of table 4 in the revised version of the manuscript.

4. There were no significant relationships between diagnosis delay and the risk of amputation (Table 4). However, the authors mentioned that "early diagnosis is essential" in Abstract (Page 3, line 65).

RESPONSE:

Thank you for this important and thoughtful observation. We agree that, strictly speaking, the variable "Diagnosis Delay" was not significantly associated with amputation outcome in our cohort, as shown in Table 4. However, our intention was to convey a broader clinical implication based on two findings: **(1) disease duration after diagnosis**, because earlier diagnosis may facilitate earlier management and treatment initiation, thereby potentially reducing disease duration; and **(2) Rutherford classification**, because earlier diagnosis may enable earlier management and help prevent progression to a higher Rutherford category. **First**, as shown in Table 4, disease duration after diagnosis was significantly associated with amputation outcome ($p < 0.05$). The longer the disease duration, the higher the risk of major amputation. **Second**, as shown in Table 5, all patients in Rutherford category 4 (rest pain) avoided amputation, whereas those in category 6

(gangrene) exhibited a significantly higher-than-expected frequency of major limb amputation ($p < 0.001$).

The original wording in the Abstract may have been too strong and may have implied a direct statistical relationship that was not demonstrated by our data. Therefore, to avoid overstatement and to align the text more precisely with the study findings, we have revised the Abstract and Conclusion sections accordingly. The following are the revised statements:

Abstract:

Prompt recognition, early management, and stringent smoking cessation measures are imperative to slowing disease progression.

[page 2, lines 42–43 in the unmarked version]

Conclusion:

Given the severe consequences of BD, including limb amputation and employment termination, prompt recognition and early management may help reduce progression to advanced ischemia, superimposed infection, and tissue loss.

[pages 21–22, lines 411–413 in the unmarked version]

Lastly, we sincerely thank you all for the time and effort invested in reviewing this manuscript. We greatly value your constructive feedback, which has helped improve the clarity and rigor of this work.

Sincerely,

Mohammed Z. Allouh
Professor
College of Medicine and Health Sciences, UAEU, UAE
Email: m_allouh@uaeu.ac.ae
On behalf of all authors
