# Supplementary material for: Clinical outcomes of critical limb ischemia in Buerger disease: A contemporary analysis from an academic referral center in Jordan
Source: PLoS One. 2026 Jun 10;21(6):e0351071. doi: 10.1371/journal.pone.0351071 (PMC13252764; doi:10.1371/journal.pone.0351071)
Supplement: Peer review history

# Peer review: Decision Letter 0

**DOI:** 10.1371/journal.pone.0351071.r001

**Authors:** Farina Binti Mohamad Yusoff

22 Feb 2026

PONE-D-25-59692

Clinical Outcomes of Critical Limb Ischemia in Buerger Disease: A Contemporary Analysis from an Academic Referral Center in Jordan

PLOS One

Dear Dr. Allouh,

Thank you for submitting your manuscript to PLOS ONE. After careful consideration, we feel that it has merit but does not fully meet PLOS ONE’s publication criteria as it currently stands. Therefore, we invite you to submit a revised version of the manuscript that addresses the points raised during the review process.

==============================

Thank you for your patience while we complete the manuscript reviews. We truly appreciate your understanding and constructive feedback.

We are writing to notify you that the number of reviews required for this manuscript has been received.

Please address all comments by the reviewers, including the comments in the attachment.

==============================

Please submit your revised manuscript by Apr 08 2026 11:59PM. If you will need more time than this to complete your revisions, please reply to this message or contact the journal office at plosone@plos.org. When you're ready to submit your revision, log on to https://www.editorialmanager.com/pone/ and select the 'Submissions Needing Revision' folder to locate your manuscript file.

Please include the following items when submitting your revised manuscript:

- A letter that responds to each point raised by the academic editor and reviewer(s). You should upload this letter as a separate file labeled 'Response to Reviewers'.
- A marked-up copy of your manuscript that highlights changes made to the original version. You should upload this as a separate file labeled 'Revised Manuscript with Track Changes'.
- An unmarked version of your revised paper without tracked changes. You should upload this as a separate file labeled 'Manuscript'.

-->If you would like to make changes to your financial disclosure, please include your updated statement in your cover letter. Guidelines for resubmitting your figure files are available below the reviewer comments at the end of this letter.

If applicable, we recommend that you deposit your laboratory protocols in protocols.io to enhance the reproducibility of your results. Protocols.io assigns your protocol its own identifier (DOI) so that it can be cited independently in the future. For instructions see: https://journals.plos.org/plosone/s/submission-guidelines#loc-laboratory-protocols. Additionally, PLOS ONE offers an option for publishing peer-reviewed Lab Protocol articles, which describe protocols hosted on protocols.io. Read more information on sharing protocols at https://plos.org/protocols?utm_medium=editorial-email&utm_source=authorletters&utm_campaign=protocols.

We look forward to receiving your revised manuscript.

Kind regards,

Farina Binti Mohamad Yusoff, MBBS, Ph.D, FSVM

Academic Editor

PLOS One

Journal Requirements:

When submitting your revision, we need you to address these additional requirements.

1. Please ensure that your manuscript meets PLOS ONE's style requirements, including those for file naming. The PLOS ONE style templates can be found at

https://journals.plos.org/plosone/s/file?id=wjVg/PLOSOne_formatting_sample_main_body.pdf and https://journals.plos.org/plosone/s/file?id=ba62/PLOSOne_formatting_sample_title_authors_affiliations.pdf

2. Your ethics statement should only appear in the Methods section of your manuscript. If your ethics statement is written in any section besides the Methods, please delete it from any other section.

3. Thank you for stating the following financial disclosure:

This study was partially supported by a UPAR grant to M. Z. Allouh from the United Arab Emirates University (Grant code: G00004977, Fund no. 12M219).

Please state what role the funders took in the study. If the funders had no role, please state: "The funders had no role in study design, data collection and analysis, decision to publish, or preparation of the manuscript."

If this statement is not correct you must amend it as needed.

Please include this amended Role of Funder statement in your cover letter; we will change the online submission form on your behalf.

4. We note that Figure(s) 1, 2 in your submission contain copyrighted images. All PLOS content is published under the Creative Commons Attribution License (CC BY 4.0), which means that the manuscript, images, and Supporting Information files will be freely available online, and any third party is permitted to access, download, copy, distribute, and use these materials in any way, even commercially, with proper attribution. For more information, see our copyright guidelines: http://journals.plos.org/plosone/s/licenses-and-copyright.

We require you to either (1) present written permission from the copyright holder to publish these figures specifically under the CC BY 4.0 license, or (2) remove the figures from your submission:

a. You may seek permission from the original copyright holder of Figure(s) 1, 2 to publish the content specifically under the CC BY 4.0 license.

We recommend that you contact the original copyright holder with the Content Permission Form (http://journals.plos.org/plosone/s/file?id=7c09/content-permission-form.pdf) and the following text:

“I request permission for the open-access journal PLOS ONE to publish XXX under the Creative Commons Attribution License (CCAL) CC BY 4.0 (http://creativecommons.org/licenses/by/4.0/). Please be aware that this license allows unrestricted use and distribution, even commercially, by third parties. Please reply and provide explicit written permission to publish XXX under a CC BY license and complete the attached form.”

Please upload the completed Content Permission Form or other proof of granted permissions as an "Other" file with your submission.

In the figure caption of the copyrighted figure, please include the following text: “Reprinted from [ref] under a CC BY license, with permission from [name of publisher], original copyright [original copyright year].”

b. If you are unable to obtain permission from the original copyright holder to publish these figures under the CC BY 4.0 license or if the copyright holder’s requirements are incompatible with the CC BY 4.0 license, please either i) remove the figure or ii) supply a replacement figure that complies with the CC BY 4.0 license. Please check copyright information on all replacement figures and update the figure caption with source information. If applicable, please specify in the figure caption text when a figure is similar but not identical to the original image and is therefore for illustrative purposes only.

5. If the reviewer comments include a recommendation to cite specific previously published works, please review and evaluate these publications to determine whether they are relevant and should be cited. There is no requirement to cite these works unless the editor has indicated otherwise.

6. Please review your reference list to ensure that it is complete and correct. If you have cited papers that have been retracted, please include the rationale for doing so in the manuscript text, or remove these references and replace them with relevant current references. Any changes to the reference list should be mentioned in the rebuttal letter that accompanies your revised manuscript. If you need to cite a retracted article, indicate the article’s retracted status in the References list and also include a citation and full reference for the retraction notice.

[Note: HTML markup is below. Please do not edit.]

Reviewers' comments:

Reviewer's Responses to Questions

**Comments to the Author**

1. Is the manuscript technically sound, and do the data support the conclusions?

The manuscript must describe a technically sound piece of scientific research with data that supports the conclusions. Experiments must have been conducted rigorously, with appropriate controls, replication, and sample sizes. The conclusions must be drawn appropriately based on the data presented.

Reviewer #1: Yes

Reviewer #2: Yes

**********

2. Has the statistical analysis been performed appropriately and rigorously?

Reviewer #1: I Don't Know

Reviewer #2: Yes

**********

3. Have the authors made all data underlying the findings in their manuscript fully available?

The PLOS Data policy requires authors to make all data underlying the findings described in their manuscript fully available without restriction, with rare exception (please refer to the Data Availability Statement in the manuscript PDF file). The data should be provided as part of the manuscript or its supporting information, or deposited to a public repository. For example, in addition to summary statistics, the data points behind means, medians and variance measures should be available. If there are restrictions on publicly sharing data—e.g. participant privacy or use of data from a third party—those must be specified.

Reviewer #1: No

Reviewer #2: Yes

**********

4. Is the manuscript presented in an intelligible fashion and written in standard English?

PLOS ONE does not copyedit accepted manuscripts, so the language in submitted articles must be clear, correct, and unambiguous. Any typographical or grammatical errors should be corrected at revision, so please note any specific errors here.

Reviewer #1: Yes

Reviewer #2: Yes

**********

5. Review Comments to the Author

Please use the space provided to explain your answers to the questions above. You may also include additional comments for the author, including concerns about dual publication, research ethics, or publication ethics. (Please upload your review as an attachment if it exceeds 20,000 characters)

Reviewer #1: 1) Page 7: Access to full data is not available by clicking the link (403 Forbidden)

2) Page 14: Lines 120-121. In theory, the clinical diagnosis of Buerger's Disease required the presence of Shionoya's 5 criteria, until the emergence of the VAS consensus criteria in 2023, which began to associate angiographic and histopathological findings.

3) Page 14: Line 122. Did the patients undergo tissue biopsy of the blood vessels?

4) Page 15: Lines 144-145. The simple advice to quit smoking has low effectiveness in the literature if it is not associated with follow-up in support groups and/or the use of pharmacological support. This may be one of the factors associated with the high amputation rate found in the cohort and should be discussed.

5) Page 16: Line 176. In the results, I observe a lack of reference to how many cases were retrospectively identified, the mechanism used to search for the cases, and, among the identified cases, how many presented complete data and in which aspects they were incomplete. How many were lost to follow-up?

6) Page 24: Lines 347-348. This goes against the common notion that smoking cessation is a key factor in the treatment of Buerger's disease." Smoking cessation not only halts disease progression but has shown to effectively reduce the risk of major limb amputations." https://doi.org/10.1016/j.jss.2024.12.034

Reviewer #2: General Comments:

The aim of epidemiological study is to investigate the clinical background and outcomes of Buerger’s disease in Jordan.

The authors have reported that (1) approximately 63% of patients underwent major or minor amputation (2) elevated CRP levels, the presence of gangrene, and the presence of limb infection are independently associated with major amputation.

The results are interesting and presented in a straightforward fashion. I have some comments for the authors.

Comments:

1. In this study, 33 (80.5%) patients had revascularization for the treatment of Buerger’s disease (Table 3). Please provide information on the patency rate of revascularization and changes in ischemic symptoms (ulcer, gangrene) during follow-up. This information helps us to understand the effectiveness of revascularization for Buerger’s disease.

2. There were no significant relationships between smoking status at enrollment and the risk of amputation (Table 4). Smoking cessation is important for preventing disease progression in Buerger’s disease. Please provide information about the effects of smoking cessation on outcomes.

3. What is the definition of “family history”? (Table 4)

4. There were no significant relationships between diagnosis delay and the risk of amputation (Table 4). However, the authors mentioned that “early diagnosis is essential” in Abstract (Page 3, line 65).

**********

6. PLOS authors have the option to publish the peer review history of their article (what does this mean?). If published, this will include your full peer review and any attached files.

If you choose “no”, your identity will remain anonymous but your review may still be made public.

**Do you want your identity to be public for this peer review?** For information about this choice, including consent withdrawal, please see our Privacy Policy.

Reviewer #1: **Yes:** Pedro Coelho Nogueira Diogenes

Reviewer #2: No

**********

[NOTE: If reviewer comments were submitted as an attachment file, they will be attached to this email and accessible via the submission site. Please log into your account, locate the manuscript record, and check for the action link "View Attachments". If this link does not appear, there are no attachment files.]

To ensure your figures meet our technical requirements, please review our figure guidelines: https://journals.plos.org/plosone/s/figures

You may also use PLOS’s free figure tool, NAAS, to help you prepare publication quality figures: https://journals.plos.org/plosone/s/figures#loc-tools-for-figure-preparation.

NAAS will assess whether your figures meet our technical requirements by comparing each figure against our figure specifications.

Attachment Submitted filename: PONE-D-25-59692_reviewer CommentsPedroDiogenes.pdf

# Author comment: Author response to Decision Letter 1

**DOI:** 10.1371/journal.pone.0351071.r002

29 Mar 2026

Response Letter

PLOS One

Manuscript ID: PONE-D-25-59692

Manuscript Title: Clinical Outcomes of Critical Limb Ischemia in Buerger Disease: A Contemporary Analysis from an Academic Referral Center in Jordan

Dear Editor and Reviewers,

We sincerely thank you for your valuable and constructive comments provided to improve this manuscript. Changes have been made in the manuscript according to the comments and suggestions. All corrections in the revised manuscript are marked by the track changes option in the copy labeled “Revised Manuscript with Track Changes”. Additionally, an unmarked version of the revised manuscript is provided in the file labeled “Manuscript”.

Finally, a detailed point-by-point response to the comments is provided herewith. Please note that your comments are in black text, and our responses are in blue.

------------------------------------------------------------------------------------------------------------------

Journal Requirements

1. Please ensure that your manuscript meets PLOS ONE's style requirements, including those for file naming. The PLOS ONE style templates can be found at

https://journals.plos.org/plosone/s/file?id=wjVg/PLOSOne_formatting_sample_main_body.pdf and https://journals.plos.org/plosone/s/file?id=ba62/PLOSOne_formatting_sample_title_authors_affiliations.pdf

RESPONSE: Done. The manuscript has been thoroughly revised to meet PLOS ONE style requirements.

2. Your ethics statement should only appear in the Methods section of your manuscript. If your ethics statement is written in any section besides the Methods, please delete it from any other section.

RESPONSE: Done. The ethical consideration statement has been included in the Methods section only. [page 4, lines 86–91 in the unmarked version]

3. Thank you for stating the following financial disclosure:

This study was partially supported by a UPAR grant to M. Z. Allouh from the United Arab Emirates University (Grant code: G00004977, Fund no. 12M219).

Please state what role the funders took in the study. If the funders had no role, please state: "The funders had no role in study design, data collection and analysis, decision to publish, or preparation of the manuscript."

If this statement is not correct you must amend it as needed.

Please include this amended Role of Funder statement in your cover letter; we will change the online submission form on your behalf.

RESPONSE: Done. The funder had no role in the study, and the statement recommended above has now been included in the “Funding” section of the manuscript.

[page 23, lines 444–445 in the unmarked version]

4. We note that Figure(s) 1, 2 in your submission contain copyrighted images. All PLOS content is published under the Creative Commons Attribution License (CC BY 4.0), which means that the manuscript, images, and Supporting Information files will be freely available online, and any third party is permitted to access, download, copy, distribute, and use these materials in any way, even commercially, with proper attribution. For more information, see our copyright guidelines: http://journals.plos.org/plosone/s/licenses-and-copyright.

We require you to either (1) present written permission from the copyright holder to publish these figures specifically under the CC BY 4.0 license, or (2) remove the figures from your submission:

a. You may seek permission from the original copyright holder of Figure(s) 1, 2 to publish the content specifically under the CC BY 4.0 license.

We recommend that you contact the original copyright holder with the Content Permission Form (http://journals.plos.org/plosone/s/file?id=7c09/content-permission-form.pdf) and the following text:

“I request permission for the open-access journal PLOS ONE to publish XXX under the Creative Commons Attribution License (CCAL) CC BY 4.0 (http://creativecommons.org/licenses/by/4.0/). Please be aware that this license allows unrestricted use and distribution, even commercially, by third parties. Please reply and provide explicit written permission to publish XXX under a CC BY license and complete the attached form.”

Please upload the completed Content Permission Form or other proof of granted permissions as an "Other" file with your submission.

In the figure caption of the copyrighted figure, please include the following text: “Reprinted from [ref] under a CC BY license, with permission from [name of publisher], original copyright [original copyright year].”

b. If you are unable to obtain permission from the original copyright holder to publish these figures under the CC BY 4.0 license or if the copyright holder’s requirements are incompatible with the CC BY 4.0 license, please either i) remove the figure or ii) supply a replacement figure that complies with the CC BY 4.0 license. Please check copyright information on all replacement figures and update the figure caption with source information. If applicable, please specify in the figure caption text when a figure is similar but not identical to the original image and is therefore for illustrative purposes only.

RESPONSE: Thank you for this important comment. We would like to clarify that the images included in Figures 1 and 2 are original patient images obtained by our team and are not reproduced from any previously published work. To fully address the journal’s copyright and licensing requirements, we have replaced the previous versions of these figures with newly prepared figures constructed in PowerPoint using only our original images and author-generated labels and layout elements. Accordingly, the revised Figures 1 and 2 do not contain third-party copyrighted material and are fully compliant with the journal’s CC BY 4.0 licensing requirements.

5. If the reviewer comments include a recommendation to cite specific previously published works, please review and evaluate these publications to determine whether they are relevant and should be cited. There is no requirement to cite these works unless the editor has indicated otherwise.

RESPONSE: There were no reviewer recommendations to cite any specific previously published works.

6. Please review your reference list to ensure that it is complete and correct. If you have cited papers that have been retracted, please include the rationale for doing so in the manuscript text, or remove these references and replace them with relevant current references. Any changes to the reference list should be mentioned in the rebuttal letter that accompanies your revised manuscript. If you need to cite a retracted article, indicate the article’s retracted status in the References list and also include a citation and full reference for the retraction notice.

RESPONSE: The reference list was prepared using Zotero software in accordance with the PLOS One guidelines. No retracted papers were cited in the reference list.

------------------------------------------------------------------------------------------------------------------

Reviewer #1

1) Page 7: Access to full data is not available by clicking the link (403 Forbidden)

RESPONSE:

Thank you for this important technical comment. We have checked access to the data, and the link is functioning properly. Screenshots are included in our attached PDF "Response to Reviewers" file as evidence.

However, access may be denied in some cases, most likely due to technical difficulties or restrictions related to geolocation or internet service providers. Therefore, in case of difficulty accessing the data, researchers may contact the corresponding author to obtain the data file. The following statement has been added to the "Avilability of data" section: The original dataset is also available from the corresponding author in case of difficulty accessing the link.

2) Page 14: Lines 120-121. In theory, the clinical diagnosis of Buerger's Disease required the presence of Shionoya's 5 criteria, until the emergence of the VAS consensus criteria in 2023, which began to associate angiographic and histopathological findings.

RESPONSE:

Thank you for this insightful comment, which raises an important point. We agree that the diagnosis of Buerger disease has evolved, and that the 2023 VAS Delphi consensus provides a more robust contemporary framework by incorporating angiographic and histopathological findings.

However, our study is a retrospective analysis of patients diagnosed between January 2015 and January 2024, and thus, the great majority of cases were identified before the publication of these updated criteria in October 2023. For this reason, our diagnostic approach necessarily reflects the standards and real-world vascular practice that prevailed during the study period.

In our setting, Shionoya’s criteria, together with characteristic angiographic findings, represented the practical and clinically accepted framework used to establish the diagnosis. This approach also allows valid comparison with the existing literature, since many previously published studies on Buerger disease have used similar criteria. Importantly, we did not rely on Shionoya’s criteria in isolation, but combined them with angiographic evidence and radiological exclusion of atherosclerotic disease to improve diagnostic specificity and minimize misclassification.

3) Page 14: Line 122. Did the patients undergo tissue biopsy of the blood vessels?

RESPONSE:

Thank you again for raising this point. As explained above, the assessment of the patients did not involve tissue biopsy of the blood vessels. Instead, their evaluation was based on radiological imaging techniques to determine the absence of atherosclerotic plaques. The term "radiological absence" has been included into the revised manuscript.

[page 5, line 100 in the unmarked version]

4) Page 15: Lines 144-145. The simple advice to quit smoking has low effectiveness in the literature if it is not associated with follow-up in support groups and/or the use of pharmacological support. This may be one of the factors associated with the high amputation rate found in the cohort and should be discussed.

RESPONSE:

We fully agree with this important and clinically relevant observation. Indeed, simple advice to stop smoking, when provided without structured follow-up, behavioral support, or pharmacological treatment, has limited effectiveness according to the literature. We agree that this may have contributed to the high amputation rate observed in our cohort and therefore merits explicit discussion in the manuscript.

This issue is particularly relevant in our setting. In Jordan, smoking cessation services remain limited, with a lack of well-established smoking cessation clinics and structured cessation programs. In addition, smoking cessation medications are generally not covered by insurance, which further restricts patients’ access to effective therapeutic support. As a result, many patients are left with advice alone, without the multidisciplinary follow-up and treatment strategies needed to address the physiological and behavioral components of nicotine dependence.

To address your valuable comment, we have now added the following paragraph to the Discussion section:

“It is important to emphasize that simple advice to quit smoking, when not accompanied by structured follow-up, behavioral support, and pharmacological treatment, has only limited effectiveness in the literature [48,49]. This may represent one of the factors contributing to the high amputation rate observed in our cohort. Effective smoking cessation usually requires structured programs that incorporate both pharmacological aids and behavioral interventions targeting the physiological and psychological aspects of nicotine dependence. Unfortunately, the healthcare system in Jordan lacks robust smoking cessation clinics, and smoking cessation medications are not generally covered by insurance, which limits patients’ access to the support and treatment necessary for successful cessation.” [pages 19–20, lines 366–374 in the unmarked version]

New References:

48. Stead LF, Buitrago D, Preciado N, Sanchez G, Hartmann-Boyce J, Lancaster T. Physician advice for smoking cessation. Cochrane Database Syst Rev. 2013;2013: CD000165. doi:10.1002/14651858.CD000165.pub4

49. Stead LF, Koilpillai P, Fanshawe TR, Lancaster T. Combined pharmacotherapy and behavioural interventions for smoking cessation. Cochrane Database Syst Rev. 2016;3: CD008286. doi:10.1002/14651858.CD008286.pub3

5) Page 16: Line 176. In the results, I observe a lack of reference to how many cases were retrospectively identified, the mechanism used to search for the cases, and, among the identified cases, how many presented complete data and in which aspects they were incomplete. How many were lost to follow-up?

RESPONSE:

We agree that the case identification process, data completeness, and follow-up availability should be described more explicitly for clarity and transparency.

In response, we have clarified in the manuscript that this retrospective study included 41 retrospectively identified eligible patients with Buerger disease and critical limb ischemia who were extracted from the electronic medical records of King Abdullah University Hospital during the study period from January 2015 to January 2024. Case identification was performed by first searching the hospital electronic medical records using the keyword “critical limb ischemia” and then further identifying patients with Buerger disease using the keyword “Buerger disease.” The diagnosis was then verified based on the predefined study diagnostic criteria.

We have also clarified data completeness. Of the 41 included patients, 9 patients had missing CRP values, whereas the remaining baseline study variables were complete for the rest of the cohort. This point has now been stated explicitly in the revised manuscript.

Regarding follow-up, we agree that this is an important issue. However, because follow-up in this retrospective cohort was irregular and many patients had intermittent consultations, the exact number of patients lost to follow-up could not be determined reliably from the available records. For this reason, we did not perform longitudinal follow-up analyses, and we have clarified this more explicitly in the manuscript as a limitation.

To improve transparency, these methodological details and the extent of missing data have now been added to the revised manuscript as follows:

Methods section – Study protocol – first sentence:

Cases were identified retrospectively from the electronic medical records of King Abdullah University Hospital affiliated with JUST between January 2015 and January 2024 by searching the keyword “critical limb ischemia” and then identifying patients with BD using the keyword “Buerger disease.” [page 5, lines 94–97 in the unmarked version]

Results section – General and clinical characteristics – first and second sentences:

A total of 41 eligible patients were retrospectively identified and included. Missing baseline data were limited to CRP values in 9 patients; all other baseline variables were complete.

[page 7, lines 158–160 in the unmarked version]

Discussion section – Limitations paragraph – sentence in bold text:

Additionally, follow-up data were often unavailable due to intermittent consultations, with gaps frequently attributable to patients’ low socioeconomic status and health insurance limitations. Therefore, the exact number of patients lost to follow-up could not be determined reliably from the available records. These incomplete and irregular data precluded the application of time-to-event analyses, such as Kaplan–Meier curves for amputation-free survival.

[page 21, lines 396–397 in the unmarked version]

6) Page 24: Lines 347-348. This goes against the common notion that smoking cessation is a key factor in the treatment of Buerger's disease." Smoking cessation not only halts disease progression but has shown to effectively reduce the risk of major limb amputations." https://doi.org/10.1016/j.jss.2024.12.034

RESPONSE:

We apologize if our original wording may have created an unintended impression. We fully agree that smoking cessation is the cornerstone of treatment in Buerger’s disease and is associated with lower rates of vascular events and amputation. This is supported by more recent

Attachment Submitted filename: Response to Reviewers.pdf

# Peer review: Decision Letter 1

**DOI:** 10.1371/journal.pone.0351071.r003

**Authors:** Farina Binti Mohamad Yusoff

21 May 2026

Clinical Outcomes of Critical Limb Ischemia in Buerger Disease: A Contemporary Analysis from an Academic Referral Center in Jordan

PONE-D-25-59692R1

Dear Dr. Mohammed Z. Allouh,

We’re pleased to inform you that your manuscript has been judged scientifically suitable for publication and will be formally accepted for publication once it meets all outstanding technical requirements.

Within one week, you’ll receive an e-mail detailing the required amendments. When these have been addressed, you’ll receive a formal acceptance letter and your manuscript will be scheduled for publication.

An invoice will be generated when your article is formally accepted. Please note, if your institution has a publishing partnership with PLOS and your article meets the relevant criteria, all or part of your publication costs will be covered. Please make sure your user information is up-to-date by logging into Editorial Manager at Editorial Manager® and clicking the ‘Update My Information' link at the top of the page. For questions related to billing, please contact billing support.

If your institution or institutions have a press office, please notify them about your upcoming paper to help maximize its impact. If they’ll be preparing press materials, please inform our press team as soon as possible -- no later than 48 hours after receiving the formal acceptance. Your manuscript will remain under strict press embargo until 2 pm Eastern Time on the date of publication. For more information, please contact onepress@plos.org.

Kind regards,

Farina Binti Mohamad Yusoff, MBBS, Ph.D, FSVM

Academic Editor

PLOS One

Additional Editor Comments (optional):

Reviewers have completed their reviews.

Your patience is greatly appreciated.

Reviewers' comments:

Reviewer's Responses to Questions

**Comments to the Author**

1. If the authors have adequately addressed your comments raised in a previous round of review and you feel that this manuscript is now acceptable for publication, you may indicate that here to bypass the “Comments to the Author” section, enter your conflict of interest statement in the “Confidential to Editor” section, and submit your "Accept" recommendation.

Reviewer #1: All comments have been addressed

Reviewer #2: All comments have been addressed

**********

2. Is the manuscript technically sound, and do the data support the conclusions?

The manuscript must describe a technically sound piece of scientific research with data that supports the conclusions. Experiments must have been conducted rigorously, with appropriate controls, replication, and sample sizes. The conclusions must be drawn appropriately based on the data presented.

Reviewer #1: Yes

Reviewer #2: Yes

**********

3. Has the statistical analysis been performed appropriately and rigorously?

Reviewer #1: Yes

Reviewer #2: Yes

**********

4. Have the authors made all data underlying the findings in their manuscript fully available?

The PLOS Data policy requires authors to make all data underlying the findings described in their manuscript fully available without restriction, with rare exception (please refer to the Data Availability Statement in the manuscript PDF file). The data should be provided as part of the manuscript or its supporting information, or deposited to a public repository. For example, in addition to summary statistics, the data points behind means, medians and variance measures should be available. If there are restrictions on publicly sharing data—e.g. participant privacy or use of data from a third party—those must be specified.

Reviewer #1: Yes

Reviewer #2: Yes

**********

5. Is the manuscript presented in an intelligible fashion and written in standard English?

PLOS ONE does not copyedit accepted manuscripts, so the language in submitted articles must be clear, correct, and unambiguous. Any typographical or grammatical errors should be corrected at revision, so please note any specific errors here.

Reviewer #1: Yes

Reviewer #2: Yes

**********

6. Review Comments to the Author

Please use the space provided to explain your answers to the questions above. You may also include additional comments for the author, including concerns about dual publication, research ethics, or publication ethics. (Please upload your review as an attachment if it exceeds 20,000 characters)

Reviewer #1: The authors made a detailed review and have adequately addressed all my comments raised in the previous round of review. They present their results in a scientifically structured form and reached conclusions that are according to their findings. In my point of view their research, once published, will contribute to the body of scientific knowledge regarding Buerger’s disease.

Reviewer #2: I would like to thank the authors for the detailed revision. I have no more comments.

**********

7. PLOS authors have the option to publish the peer review history of their article (what does this mean?). If published, this will include your full peer review and any attached files.

If you choose “no”, your identity will remain anonymous but your review may still be made public.

**Do you want your identity to be public for this peer review?** For information about this choice, including consent withdrawal, please see our Privacy Policy.

Reviewer #1: **Yes:** Pedro Coelho Nogueira Diogenes

Reviewer #2: No

**********

# Editor report: Acceptance letter

**DOI:** 10.1371/journal.pone.0351071.r004

**Authors:** Farina Binti Mohamad Yusoff

PONE-D-25-59692R1

PLOS One

Dear Dr. Allouh,

I'm pleased to inform you that your manuscript has been deemed suitable for publication in PLOS One. Congratulations! Your manuscript is now being handed over to our production team.

At this stage, our production department will prepare your paper for publication. This includes ensuring the following:

* All references, tables, and figures are properly cited

* All relevant supporting information is included in the manuscript submission,

* There are no issues that prevent the paper from being properly typeset

You will receive further instructions from the production team, including instructions on how to review your proof when it is ready. Please keep in mind that we are working through a large volume of accepted articles, so please give us a few days to review your paper and let you know the next and final steps.

Lastly, if your institution or institutions have a press office, please let them know about your upcoming paper now to help maximize its impact. If they'll be preparing press materials, please inform our press team within the next 48 hours. Your manuscript will remain under strict press embargo until 2 pm Eastern Time on the date of publication. For more information, please contact onepress@plos.org.

You will receive an invoice from PLOS for your publication fee after your manuscript has reached the completed accept phase. If you receive an email requesting payment before acceptance or for any other service, this may be a phishing scheme. Learn how to identify phishing emails and protect your accounts at https://explore.plos.org/phishing.

If we can help with anything else, please email us at customercare@plos.org.

Thank you for submitting your work to PLOS ONE and supporting open access.

Kind regards,

PLOS ONE Editorial Office Staff

on behalf of

Dr. Farina Binti Mohamad Yusoff

Academic Editor

PLOS One
